# Biomimetic Tympanic Membrane Replacement Made by Melt Electrowriting

**DOI:** 10.1002/adhm.202002089

**Published:** 2021-01-27

**Authors:** Max von Witzleben, Thomas Stoppe, Tilman Ahlfeld, Anne Bernhardt, Marie‐Luise Polk, Matthias Bornitz, Marcus Neudert, Michael Gelinsky

**Affiliations:** ^1^ Carl Gustav Carus Faculty of Medicine Center for Translational Bone, Joint and Soft Tissue Research Technische Universität Dresden Fetscherstr. 74 Dresden 01307 Germany; ^2^ Carl Gustav Carus Faculty of Medicine Department of Otorhinolaryngology Head and Neck Surgery Ear Research Center Dresden Technische Universität Dresden Fetscherstr. 74 Dresden 01307 Germany

**Keywords:** biomimicry, implants, melt electrowriting, MEW, tympanic membranes

## Abstract

The tympanic membrane (TM) transfers sound waves from the air into mechanical motion for the ossicular chain. This requires a high sensitivity to small dynamic pressure changes and resistance to large quasi‐static pressure differences. The TM achieves this by providing a layered structure of about 100µm in thickness, a low flexural stiffness, and a high tensile strength. Chronically infected middle ears require reconstruction of a large area of the TM. However, current clinical treatment can cause a reduction in hearing. With the novel additive manufacturing technique of melt electrowriting (MEW), it is for the first time possible to fabricate highly organized and biodegradable membranes within the dimensions of the TM. Scaffold designs of various fiber composition are analyzed mechanically and acoustically. It can be demonstrated that by customizing fiber orientation, fiber diameter, and number of layers the desired properties of the TM can be met. An applied thin collagen layer seals the micropores of the MEW‐printed membrane while keeping the favorable mechanical and acoustical characteristics. The determined properties are beneficial for implantation, closely match those of the human TM, and support the growth of a neo‐epithelial layer. This proves the possibilities to create a biomimimetic TM replacement using MEW.

## Graphical abstract

Melt electrowriting (MEW) is utilized for fabrication of biomimetic tympanic membrane (TM) replacements. These novel materials transfer the complete frequency range of the human speech while offering comparable mechanical properties to the human TM. Excellent biocompatibility is demonstrated using human keratinocytes. Coating of the MEW membranes with a sub‐µm collagen layer further improves the acoustic properties and facilitates cell adhesion.

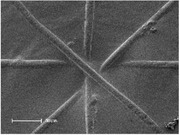

## 1 Introduction

Defects of the tympanic membrane (TM) have many causes, ranging from injuries to chronic otitis media (COM). In the U.S. 9.6 million and in Europe 5.2 million people suffer from chronically infected middle ears and about half of them struggle with yearlong hearing impairments.^[^ ^1^ ^]^ In healthy humans, the TM has a great self‐healing potential for acute minor and middle‐sized perforations. In contrast, in patients with COM the self healing capacity is diminished due to a lack of epithelial growth along the perforated border.^[^ ^2^ ^]^ Recurrent acute infections of the middle ear caused by the permament exposure to the environment evoke both, an increase in perforation size and sklerotic degeneration of the middle ear mucosa. Thus, a stable and permanent TM reconstruction is crucial for inflammation control and regeneration of the middle ear cavity. At the same time, the perfect biomechanical balance must be found between stability and the best possible vibration capability of the TM reconstruction. The clinical standard for reconstruction of the TM is an autologous graft of perichondrium, fascia or cartilage. When using perichondrium or fascia, the acoustic properties are usually adequately restored, but the reconstruction is less stable. Therefore, if pathological middle ear aeration persists, recurrent defects may easily occur.^[^ ^3^, ^4^ ^]^ However, when thin layers of autologous cartilage are used, the stability of the reconstruction is higher, so that retractions and new perforations are rare.^[^ ^5^ ^]^ Disadvantageously, the acoustic properties are significantly deteriorated, as the vibrational behavior of cartilage is different to that of perichondrium, fascia or the TM.^[^ ^3^, ^5^ ^]^

**Figure 1:**
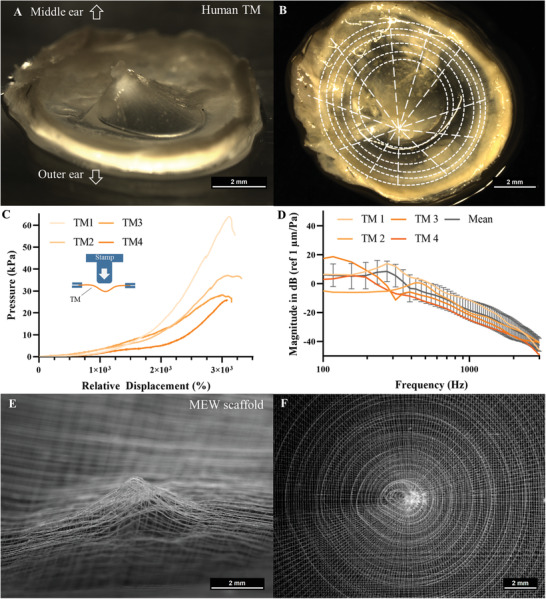
Biomimetic approach of designing a melt electrowritten TM implant. A) Stereo microscopic image of a human TM with the collapsed 3D curvature toward the middle ear. B) Top view of the TM from A) with additional sketch (white lines) to indicate the assumed circular and the radial orientation of collagen fibers within the TM. C) Measurement of the mechancical properties of a human TM with an iIllustration of the mechanical fixation and measuring concept. D) Sound transfer function frequency response curve of four human TMs with removed malleus in the test stand, measured with laser Doppler vibrometry at the umbo from the medial side. E) Porous melt electrowritten PCL scaffold mimicking the 3D curvature of the TM. F) Melt electrowritten scaffold mimicking the radial and circular collagen fiber orientation.

A crucial aspect besides the effect of employing a different material within the TM, is the preparation of autografts harvested from cartilage and perichondrium. Briefly, the tissues are cut by hand during the surgery leading to unpredictable outcomes as the thickness differs, ranging commonly from 300 up to 500 µm.^[^ ^3^ ^]^ However, the average thickness of the TM is about 100 µm and reported to be the most critical parameter of the TM.^[^ ^6^ ^]^ Therefore, a tremendous increase in thickness induces an acoustical transfer loss.^[^ ^6^ ^]^ Accordingly, a high demand exists for implant‐based strategies substituting either the entire or partly ruptured TM with replacements within the dimensions of the TM.^[^ ^7^ ^]^ The measured thickness of the healthy human TM varies, depending on the area of the TM, the inter‐individual variation and the measurement technique. According to current state, the thickness of the functionally relevant *pars tensa* region (all three layers) can range from about 30–150 µm.^[^ ^6^, ^8^, ^9^ ^]^ Moreover, replacements should resemble the overall dimensions of the TM with up to 12 mm in diameter as shown in **Figure** **1**A and support its function by closely mimicking its vibrational behavior.^[^ ^6^ ^]^ In addition, such implants need to support adhesion of epithelial cells (i.e., keratinocytes) as those cells cover the natural TM acting as a pathogen barrier.

In the past, various strategies for fabricating synthetic TM replacements were developed;^[^ ^10^, ^11^ ^],^ e.g., patches as thin as the human TM were casted from silk fibroin but lacked structural elements like the natural radial and circular collagen fiber orientation.^[^ ^12^ ^]^ These are assumed to be important for the mechanical and vibrational behavior of the human TM.^[^ ^13^ ^]^ Additive Manufacturing techniques, for example Fused Deposition Modeling (FDM), were already successfully used for the fabrication of individually sized TM, moreover, the printed scaffolds rudimentarily mimicked the collagen fiber orientation.^[^ ^14^, ^15^, ^16^ ^]^ However, the minimal feature size of this printing method is limited and the resulting membranes exhibited an overall thickness ranging from 322^[^ ^14^ ^]^ to 616 µm^[^ ^15^ ^]^ while providing a comparable vibration behavior.

Recently, Melt Electrowriting (MEW) of bioresorbable thermoplasts was introduced as novel Additive Manufacturing technique allowing the development of millimeter‐sized constructs with micrometer precision in fiber size and deposition accuracy.^[^ ^17^ ^]^ In contrast to FDM, where molten polymers are directly extruded on the printing bed, MEW uses an electrical high voltage to stabilize a molten jet at low flow rates so that it can be direct‐written onto a collector.^[^ ^18^, ^19^ ^]^ By adjustment of the applied voltage, the fiber can be deposited in a controlled manner but with a distance of several millimeters between printing bed and nozzle as otherwise a short circuit could occur. In comparison, the minimum strand size for FDM printed strands is around 100 µm,^[^ ^20^ ^]^ while MEW fibers can be fabricated with a minimum feature size of 800 nm.^[^ ^21^ ^]^ MEW can be seen as a special case of electrospinning (ES), since polymer melts are used instead of polymer solutions. The ES process has already been investigated multiple times with different materials for a possible TM reconstruction. However, the specific mechanical properties of the TM could not be reconstructed while maintaining optimum vibrational properties.^[^ ^14^, ^22^, ^23^ ^]^

By adjusting all printing parameters in the MEW process, molten polymer fibers with variety of diameters in the micrometer range can be extruded and freely stacked on top of each other.^[^ ^21^ ^]^ This enables the fabrication of thin membranes with defined architectures which can be applied to a huge variety of applications.^[^ ^24^, ^25^, ^26^, ^27^, ^28^, ^29^, ^30^, ^31^ ^]^ Its high precision together with its great degree of freedom in design makes MEW a compelling method for fabrication of a biomimetic TM implant as 3D printed constructs with exact fiber orientation and thicknesses like those in the human TM can be manufactured. Ideally, the structural and acoustic properties of the TM could be mimicked while offering a higher reconstruction stability compared to perichondrium or fascia.

In this study, we aimed to develop a synthetic TM replacement with the aid of MEW. As biomaterial polycaprolactone (PCL)^[^ ^32^ ^]^ was used as it is the best investigated biodegradable, thermoplastic polymer for MEW applications.^[^ ^29^, ^33^ ^]^ Different layer patterns were characterized concerning their acoustical and mechanical properties and it could be shown that these structures nicely mimic the vibration properties of a human TM. To ensure airtightness of the respective implants, a strategy was developed to seal the immanent micropores of the MEW scaffolds by applying a thin collagen coating. Furthermore, the cytocompatibility of the membranes with and without coating in cell culture experiments with human keratinocytes was investigated. All implemented materials were of medical grade and approved by the U.S. Food and Drug Administration (FDA) for other applications within the human body.

## 2 Results

### 2.1 Characterization of a Human TM and Biomimetic Design Strategy for Melt Electrowritten Constructs

#### 2.1.1 Experimental Characterization of a Human TM

In order to ensure the highest comparability between the human TM properties and the MEW scaffolds, the properties of both were measured in the same experimental setup (Figure S1, Supporting Information). The setup was designed to control the fixation force, which holds the specimens in place as this force was reported to have significant influence on the determined TM properties. ^[^ ^12^, ^34^ ^]^ Further, this setup was used to assess the mechanical and the vibrational properties of the TM samples and the MEW scaffolds and as such allowing conclusions between both experiments. The mechanical properties were determined by indenter measurements, instead of quasi‐static air pressure application, as these were not feasible due to the immanent scaffolds’ porosity. A human TM is shown in Figure 1A and a detailed description of the structure of the human TM can be found in Figure S2 in the Supporting Information.

The mechanical investigation of one TM is shown in Figure 1C where the pressure is plotted against the relative displacement. For force/displacement curves see Figure S3 in the Supporting Information. The TM was detached from the *malleus* and was minimally fixed within the setup in such a manner that the 3D curvature remained loose in the center with increasing stiffness toward the border. The indentation measurements were performed close to the center and the recorded pressure showed two different slopes. A lower increase at the beginning until a relative displacement of approximately 2000% was reached, and then the slope quickly enlarged. Hence, the lower slope reflected the initial relative displacement necessary to span the TM completely, which is equal to the approximately cone depth of 1.7 mm (Figure S1D, Supporting Information). Further, two main slopes could be derived from the human TM data and, as such, two bending stiffnesses, one during the spanning process with (1.97 ± 0.47) kPa and one afterward with (2.83 ± 1.00) kPa. Previous measurements with fully spanned TMs and similar elongations confirmed the measured values.^[^ ^35^ ^]^

The vibrational characteristics of the human TMs are shown in Figure 1D where the mean magnitude and standard deviation of the single sound transfer functions in the test stand are plotted against the frequency. For this purpose, the TMs were placed in the experimental setup with a simple supporting fixation, since the native shape and stability is lost after explantation. The sound transfer function was determined with a laser Doppler vibrometer (LDV; Figure S1, Supporting Information). This frequency response represents the magnitude spectrum of the investigated specimen or in response to the stimulus (100 Hz to 3 kHz). The data was calculated into dB scale with respect to the magnitude per excitation sound pressure at each frequency measurement point (µm/Pa). The measured vibration pattern was compared to the sound transfer function of TMs being naturally fixed at the *anulus fibrocartilagineus* in temporal bones (TBs) and in the test stand. A relevant measure for comparison is the first resonance frequency (fRF) of the sound transfer function with regard to the stiffness and the influence of added weight of a specimen.^[^ ^36^ ^]^ The higher the fRF in conjunction with a lower overall magnitude, the greater the stiffness. Increasing the mass mainly decreases the fRF. Because of the anatomical variances, the sound transfer function of the whole human middle ear has a variance of 20 dB at the fRF in terms of magnitude, which also includes a variance in fRF.^[^ ^37^ ^]^ For the TMs, single measurements of the sound transfer function have been performed.^[^ ^38^ ^]^ The fRF of the four measured human TMs ranged from about 150–470 Hz (Figure 1D). Regarding the TM mean (geometric mean) a target fRF of about 310 Hz for the MEW scaffolds can be derived. Because of the signal‐to‐noise ratio, the magnitude in the frequencies below 150 Hz tends to be larger in the implemented setup, while a plateauing slope to the static state (0 Hz) should be seen. The data from Zahnert et al.^[^ ^39^ ^]^ (fRF at about 300 Hz) and De Greef et al.^[^ ^38^ ^]^ (fRF at about 250 and 700 Hz) confirm our findings, despite the large variance in results, which could be caused by anatomical variance or pathological reasons. Higher reported values of the fRF could be based on a stronger fixation of the samples as this induces a higher bending stiffness.^[^ ^12^ ^]^

#### 2.1.2 Design of Melt Electrowritten Scaffolds

The *pars tensa* makes up the largest part of the human TM and therefore its structure is often used as a reference design for TM replacements (Figure 1B; Figure S2A, Supporting Information).^[^ ^14^, ^15^, ^40^ ^]^ It exhibits a layered structure with a mucosal layer on the medial side, two collagen fiber layers in the center and an epidermal layer on the lateral side. The inner collagen fiber layer consists of circumferential fibers and the outer layer of radial collagen fibers.^[^ ^41^, ^42^ ^]^ This substructure of collagen fibers plays an important role for the sound transmission ^[^ ^6^, ^13^ ^]^ A detailed description of the TM is shown in Figure S2 in the Supporting Information.

The three dimensional curvature as well as the orientation of the collagen fibers have been initially mimicked by employing melt electrowriting of PCL, shown in Figure 1E,F. Although this demonstrates, that the MEW technique allows printing of TM analogues, several parameters such as the influence of the fiber diameter, the number of layers, the layer‐to‐layer orientation and the fiber spacing needed to be studied in advance. To investigate the influence of these scaffold parameters on the mechanical and vibrational behavior in a systematic manner, a flat membrane design with a simpler structure of linear aligned fibers was chosen. Such membranes are easier to fabricate, more conclusive to evaluate and fulfill, besides handling, stability and function, the surgical needs (e.g., cutting into shape and size due to anatomical variance, availability, time saving and reduction of morbidity), too.^[^ ^43^ ^]^ For the investigation of the different parameters, two PCL fiber diameters of 10 and 15 µm were chosen as both allow the fabrication of different scaffold thicknesses within the range of the human TM (30–150 µm). Scaffolds with two different layer orientations (45° and 90°), three different number of layers (4, 6, and 8 layers) and two different fiber spacings (150 and 250 µm) were printed. The minimum distance for melt electrowritten fibers of 15 µm diameter was found to be 150 µm. Therefore, a fiber spacings of 150 and 250 µm was chosen for evaluation of the influence of the fiber spacing on the mechanical and vibrational properties of the scaffolds. Due to attraction of the fibers to one another, the real fiber spacing average is slightly lower. Three samples per condition with 20 measure points each were averaged in **Table** **1**. By multiplying the number of layers with the nominal fiber diameter the maximum scaffold thickness at the fiber intersection was estimated.

**Table 1:** Display of the different investigated scaffold designs. The abbreviations name the design parameters in the following order: 1st the number of layers, 2nd the layer‐to‐layer orientation, 3rd the fiber diameter and 4th/5th the fiber spacing (intended and real). Additionally, the estimated maximum thickness of the scaffolds at the fiber intersections is given, calculated by multiplication of number of layers with the nominal fiber diameter, 10 or 15 µm, respectively

Abbreviation	Number of layers	Layer‐to‐layer o. [°]	Fiber diameter [µm]	Fiber spacing [µm] as in the G‐code	Real fiber spacing [µm]	Maximum approx. thickness [µm]
4L90d10w150	4	90	10.5 ±0.9	150	151.1 ±9.8	40
6L90d10w150	6	90	10.5 ±1.0	150	150.4 ±9.3	60
8L90d10w150	8	90	10.7 ±1.8	150	143.8 ±8.4	80
4L45d10w150	4	45	10.2 ±0.9	150	145.1 ±9.3	40
6L45d10w150	6	45	10.0 ±0.6	150	147.5 ±6.4	60
8L45d10w150	8	45	10.4 ±0.7	150	146.4 ±4.9	80
4L90d15w150	4	90	15.6 ±0.9	150	147.4 ±4.4	60
6L90d15w150	6	90	15.0 ±1.2	150	150.1 ±10.3	90
8L90d15w150	8	90	15.0 ±2.0	150	153.1 ±17.3	120
4L45d15w150	4	45	15.0 ±0.9	150	146.1 ±12.8	60
6L45d15w150	6	45	15.1 ±0.9	150	151.6 ±9.5	90
8L45d15w150	8	45	15.4 ±0.8	150	149.5 ±15.9	120
4L90d10w250	4	90	10.1 ±0.8	250	247.2 ±12.6	40
6L90d10w250	6	90	10.5 ±1.1	250	244.9 ±12.0	60
8L90d10w250	8	90	10.7 ±1.8	250	248.2 ±11.1	80
4L45d10w250	4	45	10.8 ±0.7	250	242.8 ±17.9	40
6L45d10w250	6	45	10.3 ±0.9	250	248.1 ±8.7	60
8L45d10w250	8	45	10.4 ±0.9	250	246.5 ±13.7	80

**Figure 2:**
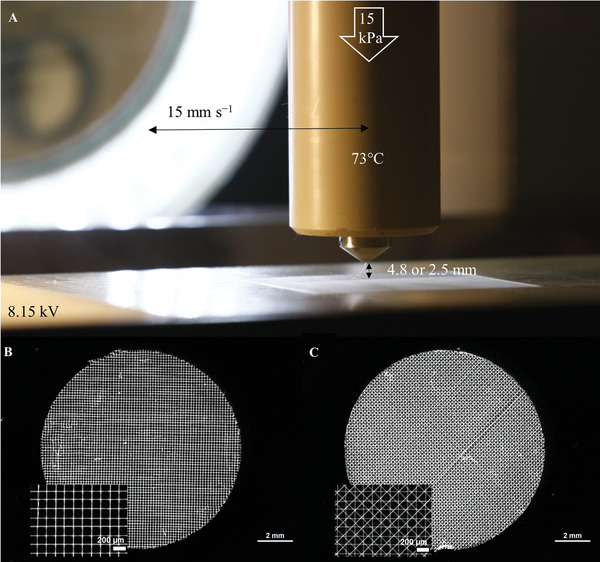
A) Photo showing the melt electrowriting process with the used printing parameters. B,C) Microscopic images of the chosen designs (90° and 45° layer‐to‐layer orientation) for investigations upon the acoustical and mechanical interplay of different scaffold parameters. The insets show cutout enlargements.

Final printing parameters were a melt temperature of 73 °C, a printing speed of 15 mm s^−1^, a voltage of 8.15 kV and a pressure of 15 kPa. Fibers with a diameter of 15 µm were printed with a distance of 2.5 mm between needle outlet and printing bed, fibers with a diameter of 10 µm with a printing height of 4.8 mm. All samples were microscopically studied to control the fiber diameter prior to further experiments. **Figure** **2**A exemplarily shows the MEW process and in Figure 2B,C examples of the MEW scaffolds with layer‐to‐layer orientations of 45° and 90° are shown.

### 2.2 Characterization of Mechanical Properties

**Figure 3:**
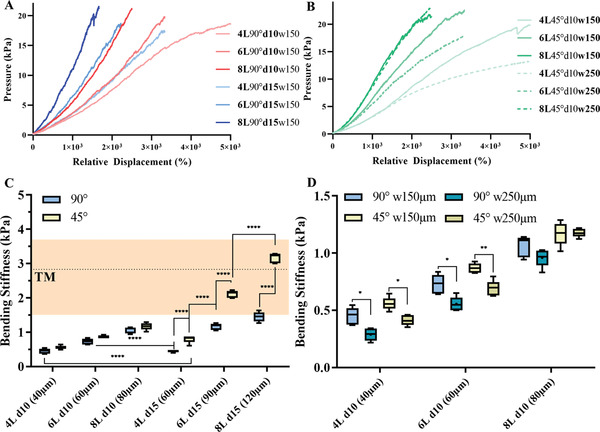
Investigation of the mechanical response of different scaffold designs. A) Pressure/relative displacement curves of fiber diameters of 10 and 15 µm and of different number of layers (4, 6, 8) at a layer‐to‐layer orientation of 90°. B) The effect of an increase of the fiber spacing from 150 to 250 µm for both layer‐to‐layer orientations and a fiber diameter of 10 µm, additionally for 4, 6, and 8 layers, is demonstrated. C) The bending stiffness of all designs is shown depending on the number of layers. The range of the measured bending stiffness of the investigated TM is highlighted in orange, the dotted line indicates the average bending stiffness of the fully spanned TM. D) The bending stiffness is ordered depending on the average scaffold thickness (*n* = 5, ± standard deviation).

The experimental setup (see 2.1.1) could be equipped to a uniaxial measuring system and pressure/relative displacement tests were performed to investigate the correlation between number of layers, fiber diameter, layer‐to‐layer orientation and fiber spacing (**Figure** **3**A,B). A rounded stamp with a diameter of 3 mm was driven with a speed of 1 mm·min^–1^. Driving force and contact area of the stamp defined the applied pressure. Displacement at the middle of the scaffold was measured and was set in relation to the scaffold thickness (relative displacement). Comparison between scaffold designs were performed by transforming the acquired data into pressure/relative displacement curves. The steepest slope of each measurement, which was then defined as bending stiffness. Pressure/relative displacement curves of scaffold designs with 4 to 8 layers and fiber diameters of 10 and 15 µm are displayed in Figure 3A. The influence of the different fiber spacing (150 and 250 µm, respectively) are presented in Figure 3B. Each scaffold was elongated by 2 mm, which resulted in high relative displacement as the thicknesses of the scaffolds ranged from (40‐120) µm. A comparison of the bending stiffnesses of the different designs is displayed in Figure 3C,D, together with the measured bending stiffness of the investigated TM. Four measurements were performed with one TM exhibiting an average bending stiffness of (2.0 ±0.5) kPa for the initial spanning process and afterward of (2.8 ± 1.0) kPa. An overview of all measured bending stiffnesses of the scaffolds can be found in **Table** **2**. The complete acquired force/displacement curves are displayed in Figure S4 in the Supporting Information.

**Table 2:** Summary of measured bending stiffness (B. s.) of all designs

Design	B. s. [kPa]	Design	B. s. [kPa]	Design	B. s. [kPa]
4L45°d10w150	0.56 ± 0.06	4L45°d15w150	0.8 ± 0.1	4L45°d10w250	0.41 ± 0.04
6L45°d10w150	0.86 ± 0.04	6L45°d15w150	2.11 ± 0.1	6L45°d10w250	0.69 ± 0.07
8L45°d10w150	1.17 ± 0.1	8L45°d15w150	3.17 ± 0.15	8L45°d10w250	1.18 ± 0.04
4L90°d10w150	0.45 ± 0.07	4L90°d15w150	0.45 ± 0.03	4L90°d10w250	0.29 ± 0.05
6L90°d10w150	0.72 ± 0.08	6L90°d15w150	1.16 ± 0.09	6L90°d10w250	0.55 ± 0.06
8L90°d10w150	1.06 ± 0.09	8L90°d15w150	1.45 ± 0.14	8L90°d10w250	0.96 ± 0.08

#### 2.2.1 Influence of the Scaffold Thickness

The scaffold thickness can either be changed through a change of number of layers or by variation of the fiber diameter. Hence, scaffolds with the same number of layers, e.g. 4, but with different fiber diameters of 10 and 15 µm differ in thickness, which was 40 and 60 µm, respectively. Remarkably, the designs of 4L90d10w150 and 4L90d15w150 with thicknesses of 40 and 60 µm exhibited an identical average bending stiffness of (0.45 ± 0.07) kPa and (0.45 ± 0.03) kPa. This changed for designs with higher number of layers and thus higher scaffold thicknesses. For 6L90d10w150 the average bending stiffness was (0.72 ± 0.08) kPa whereas for 6L90d15w150 the average bending stiffness was (1.16 ± 0.09) kPa showing a significant difference, mainly because the overall thickness differed from 60 to 90 µm. This thickness difference was a 1.5‐fold increase, which was identical to the 1.5‐fold increase in bending stiffness. This was underlined further in the case of 8 layers; the average bending stiffness for 10 µm fiber diameter scaffolds was (1.06 ± 0.09) kPa and for 15 µm fiber diameter scaffolds (1.45 ± 0.14) kPa. Here, a 1.5‐fold‐thickness difference of 80–120 µm, led to an approximately 1.5‐fold increase in bending stiffness. Additionally, the bending stiffness of 10 µm fiber diameter scaffolds doubled, when the number of layers (i.e., thickness) doubled: (0.45 ± 0.07) kPa and (1.06 ± 0.09) kPa for 4 and 8 layers, respectively. Interestingly, in the case of 15 µm fiber diameter, a 2‐fold increase of thickness from 4 to 8 layers was reflected in a three times higher bending stiffness (0.45 kPa and 1.45 kPa).

The comparison of designs with the same overall thickness (6L90d10w150 and 4L90d10w150, 60 µm), but with different fiber diameters showed different bending stiffnesses with a significantly higher bending stiffness for the 6‐layered design with (0.72 ± 0.08) kPa.

#### 2.2.2 Layer‐to‐Layer Orientation

The influence of the layer‐to‐layer orientation was investigated comparing orientations of 90° and 45°; furthermore, the differences were tested in dependence of the fiber diameter (10 and 15 µm) and the number of layers (4,6 and 8 layers). In case of 10 µm fiber diameter, the scaffolds with a layer‐to‐layer orientation of 45°, average flexural moduli of (0.56 ± 0.06) kPa, (0.86 ± 0.04) kPa and (1.17 ± 0.1) kPa were observed for 4, 6, and 8 layers, respectively. As experienced before, the bending stiffness more than doubled when the number of layers doubled. Remarkably, the bending stiffness increased about 10 kPa when the layer orientation changed from 90° to 45°. In case of 15 µm fiber diameter scaffolds with a layer‐to‐layer orientation of 45°, the average bending stiffness for each additional two layers was (0.8 ± 0.1), (2.11 ± 0.1), and (3.17 ± 0.15) kPa, respectively. In this case, a 3‐fold thickness increased the bending stiffness 4‐fold. By comparison of the bending stiffness of 45° to 90° layer‐to‐layer orientated scaffolds with the same number of layers, it was remarkable to see that the orientation change almost doubled the average bending stiffness for each individual design. This was not measured for scaffolds with 10 µm fibers: here a change of layer‐to‐layer orientation increased the bending stiffness by approximately 10 kPa.

#### 2.2.3 Fiber Spacing

The impact of varying the fiber spacing from 150 to 250 µm was exemplarily tested with scaffolds with the design of 90° and 45° layer‐to‐layer orientation, 10 µm fiber diameter consisting of 4, 6, or 8 layers, respectively. The comparison of the mechanical responses of scaffolds with identical thicknesses and layer‐to‐layer orientation but with a fiber spacing of 250 µm compared to 150 µm exhibited similar relative displacement at lower pressure values. Hence, a decrease of the bending stiffness of (16.25 ± 1) kPa was noticed for 4‐layered and 6‐layered designs. However, for the 8‐layered scaffolds, no difference in the bending stiffness was recognized (Figure 3D). Designs with a 45° layer‐to‐layer orientation showed again a higher bending stiffness when compared to 90° layer‐to‐layer orientation confirming the previous findings.

### 2.3 Characterization of Vibrational Behavior

The measurements of the vibrational behavior of human TMs were performed in TBs and in a test stand to provide comparability with the scaffolds, which were investigated in the test stand only.

**Figure 4:**
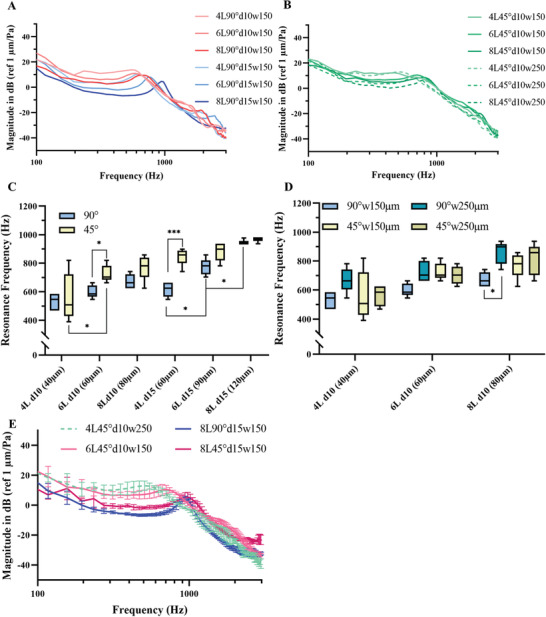
Acoustic properties of MEW scaffolds, measured with higher fixation forces. A) Comparison of sound transfer function mean curves (for standard deviation see Figure S7 in the Supporting Information) between scaffolds with 10 and 15 µm fiber diameter, 4 to 8 layers, 90° layer orientation, 150 µm fiber spacing. B) Comparison of sound transfer function mean curves (for standard deviation see Figure S7 in the Supporting Information) between 150 and 250 µm fiber spacing scaffolds with 4 to 8 layers, 45° layer orientation and 10 µm fiber diameter, C,D) Box plots of the first resonance frequencies and E) Sound transfer function mean curves (*n* = 5) and standard deviations for the scaffold designs with similar bending stiffness, as described in 2.3.4.

The test stand allowed variable fixation force in terms of radial tension caused by the fixation mechanism at the specimen rim. With the lowest applied fixation force, the scaffolds should be in a nearly unstressed state and only been hold down to the test stand. For a direct comparison to the TM in the test stand, the lowest applied fixation force was used for the scaffold measurements (see Figure S5C,D in the Supporting Information) as the human TM in its natural shape is also attached to the *anulus fibrocartilagineus* without any additional radial stress. The stable cone shape is provided by additional attachment to the *malleus* (Figure S1B, Supporting Information). In comparison to the mechanical testing, the fixation force during vibration measurements was lower since slipping of the meshes did not have to be prevented. Further, all mechanically characterized designs as noted in Table 2 were investigated. The magnitude of the vibration depending on the excitation frequency for different number of layers and fiber diameters is shown in **Figure** **4**A and for different fiber spacing in Figure 4B. The complete measured data can be found in Figure S5 in the Supporting Information. Due to the porosity of the scaffolds, a low signal‐to‐noise ratio below about 300 Hz was occured, leading to a larger magnitude. The fRF of some scaffold desgins tended to aggravate in that regime an exact determination of the fRF. By slightly increasing the fixation force the fRF were shifted to higher frequencies with a better signal‐to‐noise ratio.

We expected a correlation between the mechanically measured bending stiffness and the fRF, as a lower bending stiffness should lead to a decrease in fRF and an increase in magnitude.^[^ ^39^ ^]^

In general, the comparison between the scaffolds in regard to fRF and bending stiffness showed qualitatively similar trends, being described in the following paragraphs. All fRFs are depicted in Figure 4C,D. The vibrational behaviour of the scaffolds with similar bending stiffness as the TM (6L45°d15w150, 8L90°d15w150, and 4L45°d10w250) are shown in Figure 4E. The standard deviation of the frequency response curves are shown. Despite the similar stiffness in bending, the sound transfer functions differ mainly in fRF and magnitude because of the different thicknesses. Increased thickness results in an increased higher stiffness (higher fRF and lower magnitudes). The range and characteristics of the standard deviation are comparable with the curves in Figure 4A,B, and were omitted there for better readability (see Figure S7 in the Supporting Information).

#### 2.3.1 Scaffold Thickness

Like contemplated in 2.2.1, the scaffold thickness depends on two parameters, the fiber thickness and the number of layers. Overall, an increase in scaffold thickness increased the fRF independently of the scaffold parameters. For the 45° scaffolds, the difference between 4 and 6 layers was smaller, compared to the difference in fRF between 6 and 8 layers. Furthermore, the comparison of scaffolds with the same thickness and layer‐to‐layer‐orientation (4L90d15w150 and 6L90d10w150, as well as, 4L45d15w150 and 6L45d10w150) but with different fiber diameters showed on average about the same fRF range for the 90° scaffolds, with a higher mean for the 15 µm fiber diameter. For the related 45° scaffolds, the fRF range was also higher for the 15 µm fiber scaffolds. We assume that the fiber diameter dominantly contributes to the fRF increase, compared to the layer number. This seems to be the case, when the fibers are more interconnected like for the 45° scaffolds, compared to the 90° scaffolds.

#### 2.3.2 Layer‐to‐Layer Orientation

Changing the layer orientation from 90° to 45° showed a tendency of higher fRFs for the 45° oriented scaffolds with 150 µm fiber spacing. In contrary, a higher or similar fRF for the 90° oriented scaffolds with 250 µm fiber spacing could be seen, compared to the 45° ones.

Possible influences are discussed in paragraph 3.1.2.

#### 2.3.3 Fiber Spacing

For the scaffolds with 250 µm fiber spacing, the 90° scaffolds showed an increase in fRF compared to those with 150 µm spacing. This seems unexpected because the stiffness should decrease with fewer fibers per equal acoustically excited scaffold area. For comparison, the bending stiffness decreased with bigger fiber spacing. Compared to the bending stiffness, the differences for the 45° scaffolds did not come out as distinctively for the fRF between the scaffolds with 150 and 250 µm fiber spacing.

### 2.4 Application of Collagen and Effects of Sterilization

It was important to produce an air‐pressure tight membrane and to investigate the influence of gamma sterilization on the scaffolds for a possible transition to clinical application. The design 4L45°d10w250 showed the best compliance in the vibrational measurements and together with its fast manufacturing compared to other designs it was selected for further investigations. Collagen type I was chosen to seal the micropores for several reasons; it is secreted as main extracellular matrix component during the acute healing phase of the human TM, after myringotomy and infection.^[^ ^44^, ^45^ ^]^ It is easily accessible and its rather large fibrils were expected to best close the large pores of the scaffolds.

**Figure 5:**
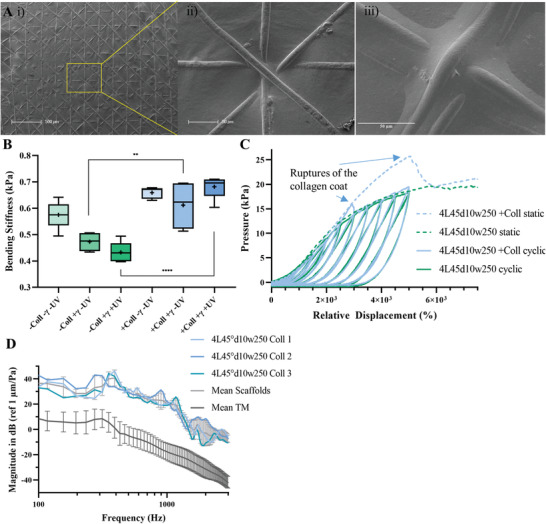
Application of collagen type I to the scaffold design 4L45°d10w250 and mechanical characterization. Ai) It is demonstrated that collagen covers the complete scaffold surface achieving an airtight structure. Even small areas revealing more complex pore geometries were filled with collagen as shown in (Aii) and (Aiii). B) The change of the mechanical properties through the application of gamma‐ sterilization, ultraviolet radiation w/ and w/o collagen coating. Static (*n* = 5, ± standard deviation) and cyclic loading measurements were performed for scaffolds w/ and w/o collagen, w/o any sterilization method. C) The response of 4L45d10w250 w/ and w/o collagen toward static and cyclic mechanical loading is depicted. D) The sound transfer function mean curves of collagen coated scaffolds and the mean (*n* = 3) and standard deviation in comparison to the mean human TM sound transfer function in the test stand is shown. The mean first resonance frequency is at about 370 Hz.

Before collagen was applied to the scaffold, the PCL meshes were incubated in 1 m NaOH for three hours to enhance the hydrophilicity of the polymer. Afterwards, the scaffolds were washed thoroughly with ddH_2_O and collagen was molded into the meshes. Therefore, acidic collagen solution was dispersed on top of the scaffolds and allowed to gel at 37 °C. Subsequently, the redundant hydrogel was skimmed with filter paper and the scaffold left for drying. The collagen covered the complete printed scaffold area, as seen in **Figure** **5**Ai with a height of approximately 300–400 nm (Figure S8, Supporting Information) and was stable in water and cell culture medium for 2 weeks. Sealing with collagen even worked for the smallest gaps at the intersection points of the PCL fibers, as displayed in Figure 5Aii. The sealing with collagen worked for different designs, too, which can be seen in Figure 5Aiii. Interestingly, higher collagen concentrations like 5 mg∙mL^–1^ or a repetition of the sealing procedure did not alter the thickness of the collagen layer markedly. During the drying of the collagen, the collagen layer induced tension into the MEW mesh and folding as well as warping could occur, especially after cutting the meshes with a punch. To provide conditions for reproducible mechanical and vibration measurements, the contact face of the experimental setup was wetted with deionized water. On the one hand, this led to a relaxation of the folding and warping of the meshes, on the other it acted as an additional adherence of the meshes. Hence, the employment of water increased the fixation force applied to the scaffolds, so that pure PCL scaffolds revealed an increase in bending stiffness from (0.41 ± 0.04) kPa to (0.58 ± 0.05) kPa. For scaffolds with collagen coating, the average bending stiffness increased to (0.66 ± 0.02) kPa. This change of the mechanical properties through the application of collagen and wetting with water is shown in Figure 5Bi.

Additionally, the effect of gamma sterilization (15 kGy) was investigated to determine the influence of possible scissions of polymer molecular chains on the thin MEW scaffolds.^[^ ^46^ ^]^ In agreement with previous studies,^[^ ^46^ ^]^ the average bending stiffness of scaffolds w/o and w/ collagen decreased to (0.47 ± 0.03) kPa and (0.61 ± 0.09) kPa, respectively. As the collagen application occurred prior to the sterilization, the mechanical strength of collagen was decreased too as described elsewhere.^[^ ^47^, ^48^ ^]^ Further, it was mentioned that crosslinking of the collagen coating could counteract and further increase the mechanical strength of the coating.^[^ ^47^, ^49^ ^]^ Collagen can be crosslinked by various methods.^[^ ^50^ ^]^ Here, collagen‐coated scaffolds were crosslinked with UV‐light due to its relative simple handling.^[^ ^51^ ^]^ Hence, scaffolds were exposed to UV light for 2 h after gamma irradiation and the crosslinking of the collagen raised the average bending stiffness to (0.68 ± 0.04) kPa.

#### 2.4.1 Viscoelastic Behavior

Ideally, materials for TM replacement should withstand pressures several times and show good recoverability. For the assessment of the printed (and coated) MEW scaffolds, cyclic loads were applied to 4L45°d10w250 scaffolds w/ and w/o collagen. These hysteresis measurements were accomplished by loading and unloading cycles while each loading step increased the indenter depth by 500 µm. The total elongation was set to 2 mm for viscoelastic measurements, for comparison and to enhance differences, static measurements were performed with a penetration length of 3 mm and are displayed in Figure 5C. For collagen‐free scaffolds, static and dynamic pressure loading showed a similar trend. This changed for scaffolds with collagen; higher pressure was recorded at earlier relative displacements. At a relative displacement of 5000 %, which is equal to 2 mm elongation, the pressure reached a maximum and decreased afterward. Then, it behaved similar to collagen‐free scaffolds. Cyclic loading followed the trend of static loading until a relative displacement of 1000 % was reached, then the pressure decreased and the hysteresis curves followed the trend of scaffolds w/o collagen. As static and dynamic measurements exhibited a quick decrease of pressure, which was not detected for collagen‐free scaffolds, it could be concluded that the collagen layer lost its integrity at this point.

#### 2.4.2 Acoustic Evaluation of PCL‐Collagen Composite Scaffolds

Due to the sealing of the pores, the transmission of low frequencies was enhanced when compared to collagen‐free scaffolds. The sound transfer function marginally increased in terms of the fRF. This was due to the increased stiffness caused by the dry collagen layer. The addition of collagen was of negligible height when compared to the overall scaffold height. Nevertheless, the introduced tension by the drying collagen inside the scaffolds structure led to curling and an increased stiffness. Thus, the fRF for the 4L45°d10w250 scaffolds increased to about 370 Hz (see Figure 5D). Due to the inhomogeneity of the surface of the collagen coated scaffold, the frequency response curve showed additional peaks above the fRF.

**Figure 6:**
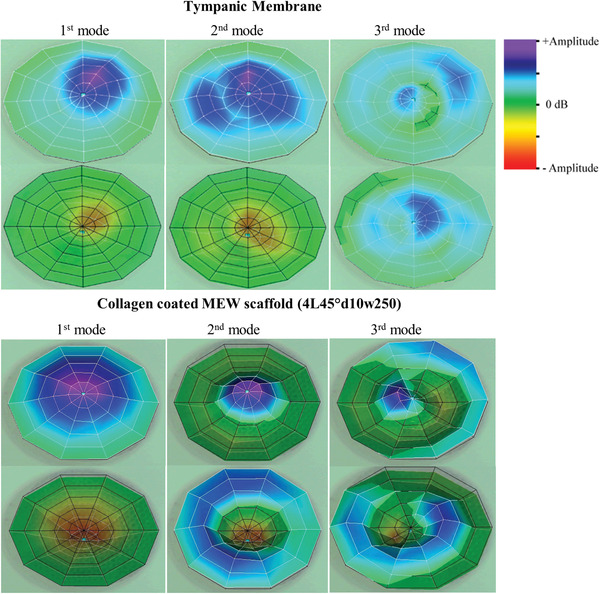
In the upper half of the Figure an image sequence showing the first vibration modes of a human tympanic membrane (*n* = 1) is depicted. The first mode is prominent at a frequency of 400 Hz, the 2nd mode at 808 Hz, and the 3rd at 1849 Hz. The lower half shows the three visible vibration modes of a flat collagen coated MEW scaffold (*n* = 1) with the design 4L45d10w250 which are prominent at 340, 800, and 1240 Hz. The first mode is expected to have the biggest contribution to the vibration of the middle ear. The maximum magnitude was decreasing with higher modes (scaled up for better visibility) as observed for the TM. The differences in the mode frequencies were caused by structural differences (e.g., curved vs. flat shape) and natural variations. The higher mode frequencies should provide a bigger variance than the first mode.

**Figure** **6** shows the vibration modes of a TM and a collagen coated scaffold in the test stand as image sequences. The corresponding videos can be found in the Supporting Information S9. The three vibration modes were acquired with a scanning LDV. The first mode shows no nodal diameter vibration and a circular node vibration. The second mode shows two nodal circles and the third mode an additional nodal line vibration. The first mode dominantly describes the scaffold stiffness. It is expected to have the biggest contribution to the vibration of the middle ear. Thus, it is the main reference for a direct comparison between the tympanic membranes and the scaffolds. A shift in frequency of the first mode means a change in stiffness. For the anatomical variance of the TM, a range of more than 300 Hz could be determined, depending on the attachment of a malleus and the test setup. Having variations from fabrication and measurement procedures in mind, Figure 6 demonstrates that the scaffolds can be tuned to show a similar vibration behavior like the human TM. Additionally, this depends on the structural differences (cone shape with higher stiffness and higher fRF vs. flat shape). The frequency of the higher order modes provide a bigger variance, which cannot be determined for all TMs due to the damped signal after the fFR.

In comparison to the collagen coated scaffold, the vibration of the TM shows lower magnitudes and a stronger damping, aggravating the determination of the vibration shape. This is also related to the non‐symmetric 3D curvature of the tissue specimen, compared to the plain and ordered scaffold structures. The maximum magnitude was 16.86 dB (reference value is the deflection magnitude: ref µm/Pa), −2.07 dB (ref µm/Pa) and −16.2 dB (ref µm/Pa), respectively. The magnitude is the highest in the purple area and lowest in the red colored area. The magnitude was decreasing with higher modes (scaled up for better visibility).

The three visible vibration modes of a flat collagen coated MEW scaffold with the design 4L45d10w250 are shown at 340, 800, and 1240 Hz exhibiting a maximum magnitude of 75.45 dB (ref µm/Pa), 66.35 dB (ref µm/Pa) and 44.98 dB (ref µm/Pa), respectively.

### 2.5 Cell Adhesion and Proliferation

**Figure 7:**
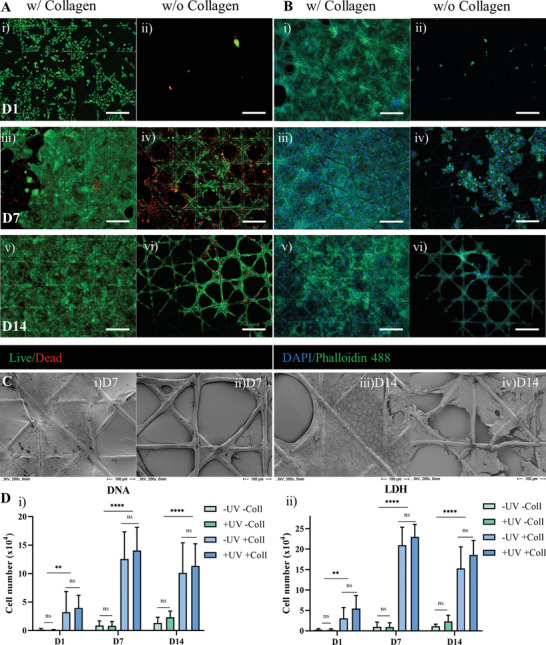
Adhesion, viability and proliferation of human keratinocytes, grown on MEW membranes with and without collagen coating for 14 days. A) Cell adhesion and viability shown with fluorescence micrographs after Live/Dead staining; viable cells appear in green and dead cells in red. B) The morphology and density of the cell layers, formed over time, is demonstrated by fluorescence microscopy, applying DAPI/Phalloidin staining (DAPI for cell nuclei/blue and Phalloidin for actin cytoskeleton/green). Scale bars in (A) and (B) represent 250 µm. SEM images confirm the results from fluorescence microscopy and show the typical, polygonal morphology of the keratinocytes, especially on collagen coated scaffolds at day 14. C) magnifications 200×. D) Cell proliferation was quantified by measurement of DNA content and intracellular LDH activity for cells grown on scaffolds w/ and w/o collagen and w/ and w/o UV treatment over 2 weeks; two individual experiments for LDH activity and DNA quantification were performed with each *n* = 3. Average *n* = 6 ± standard deviation.

Gamma sterilized 4L45°d10w250 scaffolds w/ and w/o collagen coating were seeded with cells of a human keratinocyte cell line (HaCaT) for investigation of cell adhesion on the membranes, cell viability and proliferation. Cells were seeded on top of the scaffolds and cultured over two weeks. Live/Dead staining as well as staining of nuclei and cytoskeleton with DAPI and phalloidin were performed to evaluate the cell viability and the coverage of the scaffolds after 1, 7 and 14 days, respectively (**Figure** **7**A,B). Live/Dead staining showed a high number of viable cells for all conditions but with large differences in number of initially attached cells. Staining of cytoskeleton and nuclei confirmed the finding of Live/Dead staining. Collagen‐sealed scaffolds allowed a higher initial attachment of keratinocytes and consequently latest at day 14 the scaffolds were completely covered by a cell layer. In comparison, collagen‐free PCL scaffolds showed a significantly lower number of initially attached cells, which is mostly due to the considerably lower colonizable surface area of these scaffolds. The images taken at day 7 and 14 show the beginning of cell bridging of the micropores. SEM analyses further confirmed the results of fluorescence staining (Figure 7C). Furthermore, it was investigated whether the UV light treatment for crosslinking the collagen coating (see 2.4) negatively effected the cell behavior as reported in literature.^[^ ^49^ ^]^ Here, UV light treatment had no significant effect on cell growth and coverage of the MEW meshes as depicted in the microscopic images (Figure S10, Supporting Information). A quantitative evaluation of the cell growth was performed by measurement of the cell numbers by DNA assay and cell viability by quantification of intracellular lactate dehydrogenase (LDH) activity after lysis of the cells (Figure 7D). The measurements showed a significant difference between collagen sealed and collagen free scaffolds in initial cell number on day 1. Furthermore, the crosslinked collagen coated samples demonstrated a slightly but not significantly higher initial cell number than the untreated scaffolds. This trend continued throughout the experiment. Cell numbers for collagen coated scaffolds were lower on day 14 when compared to day 7 indicating confluency of the keratinocytes at around day 7. Hence, cell growth and proliferation was enhanced by the presence of collagen and could be further improved by crosslinking of collagen.

## 3 Discussion

### 3.1 Mechanical Properties

The structural design of the synthetic membranes had significant impact on their mechanical properties as shown in Figure 3. The investigation of the thickness in general showed the expected result, that an increased thickness leads to a higher bending stiffness. Remarkable was the case when the same overall thickness was achieved by combining different numbers of layers with different fiber diameters: 4 layers of 15 µm thickness and 6 layers of 10 µm thickness. In this case, the samples with the 6 layers exhibited higher bending stiffness, which was even more surprising when the amount of material used is taken into account, since only 69% of the material of the four‐layered design was necessary to fabricate the six‐layered scaffolds. Additionally, a more detailed measurement of the thickness distribution and porosities with X‐ray microtomography could allow a closer examination of such differences in the future. Youssef et al. determined the thickness distribution for designs with a 90° or 45° fiber orientation, a fiber diameter of 10 µm and a fiber spacing of 250 µm, but with 10 or 12 layers, respectively.^[^ ^52^ ^]^ The average thicknesses determined were between 50 and 60 µm, thus significantly less than an approximated maximum thickness of 100 or 120 µm. Taking into account these corrections of the thicknesses, the determination of the bending stiffnesses could be further refined in future.

Furthermore, it has to be considered, that the stamp contacts a slightly higher number of fibers in case of the scaffolds with scaffolds with 10 µm fiber diameter (6L90°d10w150) compared to the scaffolds with 15 µm fiber diameter (4L90°d15w150), while the fiber spacing and the stamp area stay constant. For example, with a fiber spacing of 150 µm and a fiber diameter of 10 µm ca. 62 fibers fit into an area of 10×10 mm² in one layer. In case of a fiber diameter of 15 µm and a fiber spacing of 150 µm, ca. 60 fibers fit into the same area of one layer. Hence, a difference of two fibers per layer exists, which is a rather small difference compared to the overall fiber number but seems to have an impact on the very thin scaffolds with just four layers in total. Hence, in case of developing a human TM implant it could be of interest to decrease the fiber diameter further to increase the number of layer and with that probably also the bending stiffness.

Considering the layer‐to‐layer orientation at the same time, it is noticeable that the stiffness of the 4L45°d10w150 sample can be raised that of the 6L90°d10w150 scaffolds by changing the layer‐to‐layer orientation from 90° to 45°. Thus, the mechanical properties of the investigated scaffolds significantly depend on the layer‐to‐layer orientation. Comparison to literature is difficult, as MEW is a relatively new technique with a great variety of potential applications. Up to now MEW scaffolds were mainly embedded into hydrogels to increase the mechanical response to a compressive mechanical load, where the cross sectional area of the stamp was larger than that of the scaffold area.^[^ ^53^, ^54^, ^55^, ^56^ ^]^ In contrast, our mechanical experiments employed a stamp with a smaller cross sectional area than the scaffold area. We were not the first acknowledging this difference, Castilho et al.^[^ ^56^ ^]^ separated therefore their approach in congruent (larger stamp) and in‐congruent type‐loading (smaller stamp), with a significant smaller peak modulus for the in‐congruent type‐loading. Comparison of our results is further complicated as our experimental setup allowed bending of the scaffolds during the compression. Hence, a reliable comparison is not feasible.

Compared to Bas et al.,^[^ ^53^ ^]^ the induced decrease of the bending stiffness through the increase of fiber spacing was small, as they observed an almost 4‐fold decrease in their bending stiffness when they doubled the fiber spacing from 400 to 800 µm. Thus, the mechanical properties of a TM replacement could be precisely tailored by a variation of the fiber spacing.

Overall, the most significant impact on the mechanical properties of the MEW scaffolds was the layer‐to‐layer orientation and the overall thickness of the scaffolds. By variation of fiber spacing and fiber diameter, a fine‐tuning of a TM replacement became possible.

Furthermore, three bending stiffnesses of printed scaffolds are comparable to the two bending stiffnesses of the human TM as shown in Figure 3. 6L45°d15w150 and 8L90°d15w150 mimicked the initial behavior of the TM and 8L45°d15w150 matched the bending stiffness of a fully spanned TM. Hence, it was accomplished to print porous PCL scaffolds withstanding a similar mechanical load as the human TM without mimicking the collagen fibril structure.

The immament porosity of the scaffolds was succesfully filled with a thin collagen layer, which slighty increased the bending stiffness. The decrease of the bending stiffness through gamma irradiation was counteracted by UV light treatment.

In future studies it will be investigated how radial and circular fibers will perform under identical loading. Radial fibers will be unidirectional elongated and could therefore have a major impact on the mechanical properties.

### 3.2 Vibrational Properties

The addressed differences between the trend in bending stiffness and fRF might be caused by the lower deflection range of the dynamic excitation (nm to µm range, frequency dependent) in comparison to the 2 mm indentation length of the mechanical measurements. During printing, the first layer of fibers may be tensioned on the printing bed. When the scaffolds are removed after fabrication, this tension is released, which may cause a (slight) undulation or “curling”. A curled scaffold could induce a lower fRF because of a lower stiffness, while the mechanical properties would remain unaffected, as the curling is negligible over the larger elongation range of the mechanical testing. Hence, the mechanical measurements could have compensated slight scaffold differences like curling when compared to the vibrational measurements.

Additionally, differences between the mechanical and acoustical testing could be induced by different radial strains based on the fixation. For example, the scaffolds with a 250 µm fiber spacing (90°) experienced a larger strain than the 150 µm spaced scaffolds (90°), since the same radial fixation force was applied to lesser fibers. This could have increased the fRF of the 250 µm spaced scaffolds in comparison to the scaffolds with 150 µm fiber spacing. The smaller differences in fRF between the scaffolds with 150 and 250 µm fiber spacing, respectively, and a 45° fiber orientation (increased stiffness for both fiber spacing types) seem to support this assumption.

A comparison between the human TM and the 4L45°d10w250 scaffolds shows a significantly higher fRF but with a similar magnitude (Figure 4E). However, for a possible implant a higher fRF and similar magnitude could lower the hearing outcome for the patient by decreasing the transferred sound magnitude to the ossicular chain due to stiffness increases by postoperative changes like a potential retraction of the TM during the healing process.^[^ ^57^, ^58^ ^]^ Accordingly, a low fRF and a high magnitude of an optimal scaffold design mimicking the TM function are required for being able to increase the stiffness in the further development (e.g., by application of collagen, see 2.4.2) for achieving an fRF similar to that of the native human TM.^[^ ^12^ ^]^ Scaffold design 4L45°d10w250 fullfills these requirements best with a low fRF and a low bending stiffness. Despite the even lower fRF, scaffold 4L45°d10w150 was not chosen as reference, because of the high variance in vibration measurement results and the higher bending stiffness found in the mechanical testing. Another marginal point for the preferation of scaffold 4L45°d10w250 is the shorter fabrication time because of the wider fiber spacing, which could be benefitial for a production in the long‐term. The scaffold 4L45°d10w250 exhibits a comparable frequency response to the human TM in the dry state. A main difference to the measured TMs is the up to about tenfold larger magnitude. Additional loading (e.g., external influences like mass and tension because of cell ingrowth, fixation force or incorporation of a prosthesis coupler) may increase the fRF and lower the magnitude, so that the scaffold would still be in the required range of vibration behavior.

The application of collagen exhibited more resonance peaks in the higher frequency range. One reason could be the curling of the scaffolds based on the collagen application, especially when compared to the flat open fiber structure of the pure MEW scaffolds. For this first approach, the variation of about 100 Hz and 10 dB of the first resonance frequency is acceptable in comparison to the variations of the TM. The two key points of these collagen filled scaffolds are the fabrication of a closed, thin collagen infill and the small influence on resonance frequency and magnitude. However, the results of the fRF are within the range of the TM. Additional loading with water should decrease the fRF slightly, as the scaffold mass is increased.

By implementing radial and circular MEW fibers instead of grid‐like arrangement it is assumed, that a comparable acousto‐mechanical behavior will be achieved in future studies, since the presented simple grid structures already provided that.

### 3.3 Biological Response

After implantation the growth of an epithelial cell layer on the surface of the synthetic TM as it is present in the natural TM is desirable. Therefore, the adhesion and proliferation of human keratinocytes on the scaffolds was tested in vitro. As expected, initially attached number of cells was significantly higher on collagen‐coated scaffolds due to the higher colonizable surface area, leading to a completely covered scaffold surface as early as after 7 days of cultivation. Thus, collagen coating of synthetic TM implants might support the fast integration into the body. However, also collagen free scaffolds showed bridging of micropores exhibiting the capability of the pure PCL scaffolds to be covered by keratinocytes as well, just within a longer time period when compared to scaffolds coated with collagen. As MEW is a quite new technique, research just started looking into the possibility of structure induced cell growth by different orientation of MEW fibers.^[^ ^59^, ^60^ ^]^ However, an orientated growth of the keratinocytes induced by the MEW fibers was not observed in our study. In future work the cell interaction with radial and circular fibers will be investigated. It is expected, that the fiber orientation will have a minor impact on the specific orientation when a collagen coating is present, as the cells spread out uniformly over the collagen layer without aligning along the MEW fibers. Therefore, further studies with non‐coated MEW scaffolds are preferable. An initial elongation of the cells in case of radial and circular fibers, similar to that observed in the natural TM, could further improve the performance of TM implants made by MEW.

As PCL is a biodegradable polyester the MEW membranes should be remodeled over time after implantation, giving space for regeneration of the natural TM tissue. The degradation of the PCL structure should take longer than the closure by new tissue. Thus, we expect a permanent function in an acousto‐mechanical and pathogen barrier point of view, without any significant changes in vibration behavior, but this has to be further investigated in future.

The degradation kinetics will be difficult to investigate without utilizing suitable animal models as the degradation of the scaffolds highly depends on the implantation site and as such would not be completely surrounded by tissues and body liquids compared to PCL implants, e.g. used for stabilization of bone fractures.^[^ ^32^, ^61^ ^]^ Therefore data for PCL biodegradation, available in literature^[^ ^62^ ^]^ cannot be easily translated to the novel type of implant, proposed here.

Hence, it is unknown and unpredictable how a MEW printed scaffold would degrade in such a partly open and initially still inflamed environment. The degradation kinetics as well as behavior in vivo will be investigated in further studies.

## 4 Conclusion

Highly organized and biodegradable scaffolds consisting of medical grade PCL were 3D printed within the dimensions of the human TM by employing MEW. Multiple designs were created, mimicking the mechanical and the vibrational properties of the human TM. The influence of design parameters as scaffold thickness, fiber diameter, layer‐to‐layer orientation as well as fiber spacing were intensively investigated, exhibiting a strong influence of the scaffold thickness and the layer‐to‐layer orientation on the bending stiffness. The vibrational evaluation showed a suitable outcome for small fiber diameters and small number of layers as larger fiber diameter and higher number of layers increased the fRF. Additional interconnection of the fibers (as present scaffolds with 45° layer orientation) and different fiber spacing allows further adjustment options of the scaffold properties. The immanent micropores of the MEW scaffolds were sealed by collagen type I, allowing an enhanced transfer of excitation frequencies below 300 Hz. Otherwise, the scaffolds kept their properties in comparison to scaffolds without collagen coating. These observations will be incorporated into future studies with radial and circular fiber orientations to improve mechanical stability of the membranes further.

Scaffolds w/ and w/o collagen supported the growth of a neo‐epithelial layer which was investigated using human keratinocyte cell line HaCaT. Additionally, presence of the collagen coating shortened the time until confluency was reached. By crosslinking the collagen through UV light, the bending stiffness could be raised without negatively affecting the cellular response.

Epithelial cell migration and fibroblast growth within four weeks and a stable reconstruction after about 4–6 weeks are the current timeline after TM reconstruction. This needs to be investigated for the proposed MEW‐based membranes in an animal study. One of the main features should be a functional and more stable TM reconstruction based on a support material, which lasts at least three to six months after implantation, to guarantee stability during potential reinfections and to support the healing process of chronic *otitis media* without retraction or re‐perforations.

Altogether, these results show the great potential of MEW for fabrication of scaffolds as TM replacement as the properties of the natural tissue concerning thickness, mechanical and vibrational behavior can be met.

## 5 Experimental Section

### Printing and Treatment of Scaffolds

Printing was executed with a GeSiM BioScaffolder 3.1. combined with a Melt Electrowriting head (GeSiM mbH, Radeberg, Germany) utilizing a needle outlet with a diameter of 250 µm. As material medical grade polycaprolactone (PCL, Purasorb PC 12 Corbion, Amsterdam, Niederlande) was used. Overall printing temperature was finally set to 73 °C and applied voltage was 8.15 kV. For 10 µm diameter fibers a printing speed of 900 mm min^−1^ with a pressure of 20 kPa and a z‐offset of 4.8 mm and for 15 µm diameter fibers a printing speed of 1200 mm min^−1^ with a pressure of 30 kPa and a z‐offset of 4.2 mm was applied. The fiber diameter of every scaffold was checked under a light microscope; subsequently, the scaffolds were treated with 1 M NaOH for 3 h. Afterwards they were cut with circular steel punches with a diameter of 11 mm. All scaffolds were printed within 10 days with one melt to avoid major polymer aging and changes in crystallinity.^[^ ^21^ ^]^

### Collagen Coating

Collagen type I isolated from rat‐tail (Meidrix Biomedicals GmbH, Esslingen, Germany) was used. Scaffolds were immersed in 2 mL of collagen solution, diluted to a collagen concentration 1 mg mL^−1^ with 0.1 m acetic acid and incubated at 37 °C for 30 min. Excessive collagen was skimmed by carefully placing a filter paper on top of the scaffolds and scaffolds were allowed to dry in air at RT.

### Mechanical Testing

For static investigations, uniaxial compressive tests of the scaffolds and TM were performed employing a universal testing machine (Z010 equipped with a 100 N load cell, ZwickRoell, Germany). Scaffolds with a diameter of 11 mm were placed within the designed experimental setup (see Figure S1 in the Supporting Information) and pressure was applied through a stamp (diameter 3 mm). Scaffolds were fixed with a silicone ring and tightened to the same point to apply similar fixation force to each scaffold. In case of scaffolds coated with collagen, some water was added to the setup to enhance fixation. Force‐displacement curves were obtained with a velocity of 1 mm min^−1^. Pressure, relative displacement and bending stiffness were calculated. For the bending stiffness the steepest slope of each scaffold (*n* = 5) was determined.

### Vibration Measurements in the Test Stand

Before defrosting, the TMs were stored frozen and without any fixation to minimize influences on the measurements. TM specimen with an effective diameter of 8 mm were excited in a self‐constructed test stand (Figure S1, Supporting Information) at an effective diameter of 8 mm with a multi‐sinusoidal signal in the frequency range between 100 Hz and 5 kHz at a sound pressure level of about 90 dB SPL by an insert earphone (ER‐2C, Etymotic Research, USA). Scaffold specimen had a total diameter of about 11 mm. A probe Microphone (ER‐7C, Etymotic Research, USA) was placed about 1 mm in front of the scaffolds center to measure the applied sound pressure. The vibration characteristics in relation to specific structural parameters, e.g. fiber diameter, number of layers, orientation angle and fiber distance were investigated and compared to those of the human TM. For this purpose, specimen vibration velocity was measured with a laser Doppler vibrometer (CLV 700, CLV 1000 with modules M300, M050, and M003, Polytec, Germany) and then mathematically integrated to get the displacement. Mainly, the overall stiffness is relevant for the sound transfer function characteristic (magnitude and first resonance frequency shift), in our case. In detail, the mass is more relevant with higher frequency; the stiffness is by theory mostly relevant for lower frequencies, like seen in Figure S6 in the Supporting Information. The phase of the sound transfer function was manually analyzed to find the frequencies where the characteristic phase shift at the first resonance frequency peaks occurred (see Figure S6 in the Supporting Information). The variation of the results was statistically investigated within the different batches by measuring each scaffold three times at a random orientation change of at least 30° rotation to the previous measurement. Each batch consisted of five scaffolds of the same scaffold structure for being able to investigate differences in fabrication. The scaffolds were fixed in the test stand with a silicone ring and a top part. The silicone ring with a weight of 4.32 mg provided the smallest fixation force of 4.32 mg • 0.001 kg • 9.81 m/s² = 0.4 mN. Together with the top part, the weight summed up to 0,0432 + 8,3822 = 8,4254 g and a resulting fixation force of 8,4254 • 0.001 kg • 9.81 m/s² = 0,0827 N, which resultet in an about 200 times greater fixation force, shifting the fRF to higher values. In this way, the differences in fRF could be investigated more precisely because of the better signal‐to‐noise ratio (SNR) in the resulting higher frequency range. For increasing the SNR, single glass beads (P‐RETRO, Polytec, Germany) were positioned at the measurement point for the scaffold (central) and the TM (umbo region) measurements.

To achieve a better fixation of the collagen coated MEW scaffolds, the rim was wettened with a drop of water. In this way, the collagen covered scaffolds could be measured in a flat shape.

The vibration modes in Figures 4F and 5E were acquired with a scanning laser Doppler vibrometer (vibrometer controller OFV‐5000, Junction Box PSV‐400 and scanning head PSV‐400 with close‐up unit PSV‐A‐410, Polytec, Germany) without any reflective beads.

### Preparation of Tympanic Membranes and Vibration Measurements of Temporal Bones

The research related to the use of human temporal bones complied with all the relevant national regulations, institutional policies and was performed in accordance with the tenets of the Helsinki Declaration and has been approved by the authors’ institutional ethical committee at TU Dresden (study number EK 59022014).

Four adult human cadaveric TBs were used for investigating the TM function as vibration reference. The age of the four female donors was 65 – 95 years.

The TBs were comprehensively evaluated using light microscopy to exclude pathological changes until final preparation and measurement. Isotonic saline was used for storage and applied during the measurements to prevent the specimen from drying‐out.

The access to the middle ear cavity was achieved over a mastoid approach and an extended posterior tympanotomy (with facial nerve resection for a better view of the stapes footplate). The ossicles, their ligaments and tendons as well as the inner ear structures were not damaged. A hole with a diameter of about 1 mm was drilled directly into the anterior side of the bony external ear canal, opposite to the TM. A probe Microphone (ER‐7C, Etymotic Research) was inserted with the microphone tip about 1–2 mm in front of the umbo to measure the sound pressure in front of the TM.

First, a reflective foil with a size of about 0.5 mm^2^ was placed at the center of the stapes footplate for measurements of the middle ear transfer function (METF) with the LDV (see equivalent setup from Arechvo et al.^[^ ^57^ ^]^ This METF was used to characterize the TBs in case of pathological changes and to qualify them in comparison to the ASTM.^[^ ^37^ ^]^ Corresponding to the following measurement procedure, the successive degradation of the petrous bone structures took place.

For the METF measurement, an insert earphone (ER‐2C, Etymotic Research) in the ear canal applied a multi‐sinusoidal signal between 100 Hz and 5 kHz at a sound pressure level of about 90 dB SPL.

The footplate vibration velocity was measured with a laser Doppler vibrometer (CLV 700, CLV 1000 with modules M300, M050, and M003, Polytec) and then mathematically integrated to get the displacement.

After this first measurement on the petrous footplate with the intact ossicular chain, the incus was removed from the petrous bone after carefully severing the incudostapedial and incudomalleolar joints. Equivalent velocity measurement on the medial side of the umbo followed (see Figure S1C in the Supporting Information). The ablation of parts of the *pars petrosa* and *pars squamosa* of the *os temporale* took place until the TM was fully exposed from a perpendicular point of view. After measurement on the malleus handle tip, the malleus was removed and the tensed eardrum was displayed in the bony limbus. Subsequently, the eardrum including the annulus fibrosus was detached from the bony limbus. The membrane was then measured on the back side of the umbo in the test stand (see Figure S1B, Supporting Information), with the medial side facing upward.

### Cell Culture

HaCaT human keratinocytes bought from the German Collection of Microorganisms and Cell Cultures (DSMZ, Braunschweig, Germany) were cultured in Dulbecco's modified Eagle's medium (DMEM, Gibco, Darmstadt, Germany) supplemented with 10% fetal calf serum (FCS, Gibco) and 100 U mL^−1^ penicillin and 100 µg mL^−1^ streptomycin. Cells were incubated in 5% CO_2_ and 95% humidity and subsequently passaged.

### Cell Seeding

MEW scaffolds were gamma sterilized with 15 kGy before cell seeding. HaCaTs were detached from flasks by 0.25% trypsin/0.02% ethylenediaminetetraacetic acid (EDTA) solution (Gibco). Scaffolds were placed in 12 well plates with 50 µl of ddH_2_O for fixation. 50 µL of cell culture medium containing 5 × 10^4^ cells was pipetted on top of each scaffold; after 1h of incubation at 37°C 2 ml of cell culture medium was added.

### Live/Dead Staining

Cell viability was assessed by a live and dead staining using Calcein AM and Ethidium homodimer‐ 1 (EthD‐1) at day 1, 7, and 14. Calcein stained the metabolically active cells in green and EthD‐1 the dead cells in red. Imaging was performed with a fluorescence microscope (Keyence BZ 9000, Osaka, Japan).

### Scanning Electron Microscopy

Cell coverage of the different MEW scaffolds was examined at three time points (d1, d7, d14). Cells were fixed with 4% glutaraldehyde in HEPES buffer for 1h. Afterward the specimens were washed with deionized water and consecutively dehydrated in ethanol gradient series of 33%, 66% and twice 100% for 30min. Ethanol was replaced by 50% Hexamethyldisilazane (HMDS), which was subsequently exchanged with 100% HDMS. Excessive HMDS was removed from the well and the scaffolds were placed in the hood overnight for drying. Specimens were mounted on stubs, sputter coated with gold and imaged using a Zeiss DSM 982 Gemini equipped with field emission gun (Carl Zeiss, Oberkochen, Germany) as well as a Philips XL 30/ ESEM, operated in SEM mode at a voltage of 3 kV (spot size 3) with a field emission gun (*n* = 3).

### Biochemistry

DNA and LDH assays were performed to quantify cell numbers at different time points (d1, d7, d14). Cell seeded scaffolds were washed with phosphate‐buffered saline solution (PBS) and stored in a −80 °C freezer until examination. After defrosting the scaffolds cell lysis was achieved using 1% Triton X‐100 in PBS which was applied for 30 min at 37 °C including a sonification bath of 2 min. DNA content of the lysates was analysed using the QuantiFluor dsDNA Kit (Promega Corp, Madison,WI; USA) according to manufacturer´s instructions. Fluorescence at 485 nm ex/535 em was measured using a multifunction microplate reader (InfinitePro, Tecan, Männedorf, Switzerland), and relative fluorescence values were correlated with cell numbers using a cell calibration line. The activity of intracellular lactate dehydrogenase (LDH) was quantified in the same lysates using the CytoTox 96 Non‐Radioactive Cytotoxicity Assay (Promega Corp., Madison, WI, USA). To this end the lysates were further diluted with lysis buffer, the substrate solution was added and absorbance at 490 nm was monitored for 5 min. The slopes of the absorbance were correlated with the slopes measured with identically treated lysates of known cell numbers which acted as calibration curve. The measured intensity was correlated with the cell numbers by using a calibration line. The experiments were performed in triplicates and were repeated.

### Statistical Analysis

Mechanical and vibrational data were collected as mean ± standard deviation (*n* = 5) for the MEW scaffolds. For mechanical analysis, a two‐way ANOVA test with Tukey's multiple comparison method was implemented to evaluate the influence of fiber diameter, number of layers, fiber spacing and layer‐to‐layer orientation on bending stiffness (GraphPad Prism 8). Cell data (both DNA and LDH) was acquired by two individual experiments with each *n* = 3 and then two‐way ANOVA with Tukey's multiple comparison method was performed. Values of *p* < 0.05 (*) were considered significant with a stepwise increase, indicated via an additional star within the graphs (*p* < 0.01**, *p* < 0.001***, *p* < 0.0001****).

For vibration analysis, each scaffold of the pure MEW sample group (*n* = 5) was measured three times with a rotation to the previous measurement of at least about 45° to investigate differences in positioning and fixation (fixation area of the scaffolds is inhomogenous). The sound transfer function arithmetic average of the three measurements for one sample of each type of scaffold was calculated and the average of each single scaffold of one batch was calculated into dB (20•log(mean)) and used for the calculation of the sample group arithmetic average, using Origin (Originlab, USA). The fRF was determined by evaluating the transfer function (in general, sound transfer function peak below about 1 kHz) and the associated phase angle curve (increased gradient in a range of 90° to 180° at the fRF). The curve morpholohy preserving algorithm for calculating the mean curve of the TBs is described in the supplement.

## Conflict of Interest

The authors declare no conflict of interest.

## Supporting information

Supporting Information

Supplemental Video 1

Supplemental Video 2

## Data Availability

The data that supports the findings of this study are available in the supplementary material of this article.
