# Supplementary material for: Biomimetic Tympanic Membrane Replacement Made by Melt Electrowriting
Source: Adv Healthc Mater. 2021 Jan 27;10(10):2002089. doi: 10.1002/adhm.202002089 (PMC11468533; doi:10.1002/adhm.202002089)
Supplement: Supplementary file 1 — Supporting Information [file ADHM-10-2002089-s003.pdf]

© 2021 Wiley-VCH GmbH

ADVANCED HEALTHCARE MATERIALS

Supporting Information

for *Adv. Healthcare Mater.,* DOI: 10.1002/adhm.202002089

Biomimetic Tympanic Membrane Replacement Made by Melt Electrowriting

*Max von Witzleben, Thomas Stoppe, Tilman Ahlfeld, Anne Bernhardt, Marie-Luise Polk, Matthias Bornitz, Marcus Neudert, Michael Gelinsky**

Supporting Information

Biomimetic tympanic membrane replacement made by melt electrowriting

Max von Witzleben, Thomas Stoppe, Tilman Ahlfeld, Anne Bernhardt, Marie-Luise Polk, Matthias Bornitz, Marcus Neudert, Michael Gelinsky[*]

M. von Witzleben, T. Ahlfeld, Dr. A. Bernhardt, Prof. M. Gelinsky
Technische Universität Dresden, Carl Gustav Carus Faculty of Medicine, Center for Translational Bone, Joint and Soft Tissue Research, Fetscherstr. 74, 01307 Dresden, Germany
E-mail: michael.gelinsky@tu-dresden.de

T. Stoppe, Dr. M.-L.Polk, Dr. M. Bornitz, Prof. M. Neudert
Technische Universität Dresden, Carl Gustav Carus Faculty of Medicine, Department of Otorhinolaryngology, Head and Neck Surgery, Ear Research Center Dresden, Fetscherstr. 74, 01307 Dresden, Germany

Experimental setup for mechanical and vibrational investigations

The tailored test stand, used in this study, allowed a fixation of both, human TM samples and MEW scaffolds, with defined clamping force. The clamping part assembly consisted of a silicone ring and two centrically aligned clamping parts. The mechanism forces the silicone ring to stretch outwards, inducing a radial tension in the specimen. The applied force consists of the weight of the clamping parts themselves or additional force controlled by an adjusting wheel, which can be determined by measuring strain gauge half-bridges being attached at the three clamping mechanism beams. The test stand and the measurement principles are shown schematically in Figure S1.

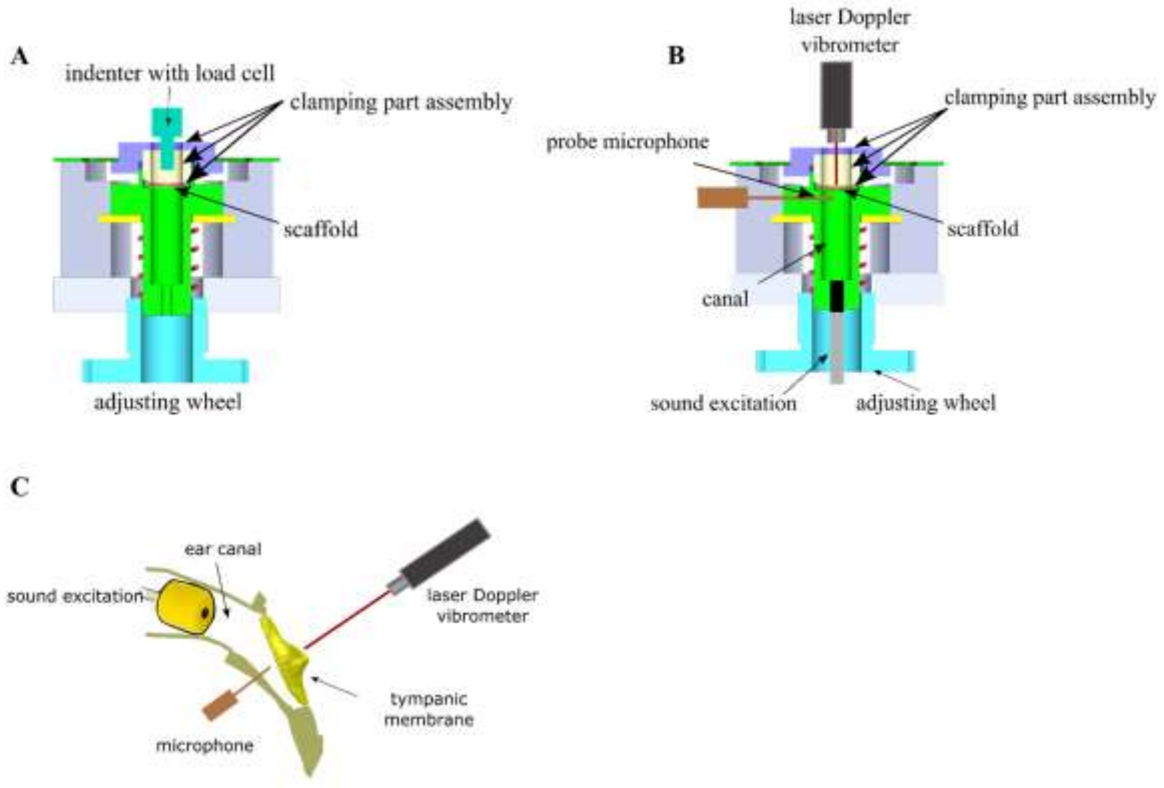

Figure S1. A) The test setup for measuring the vibration magnitude of the tympanic membranes. B) Self-constructed LDV test stand with an effective diameter of 8 mm with a multi-sinusoidal signal between 100 Hz and 5 kHz at a sound pressure level of about 90 dB SPL, generated by an insert earphone (ER-2C, Etymotic Research, USA). The scaffold specimen had a total diameter of about 11 mm. A probe Microphone (ER-7C, Etymotic Research) was placed about 1 mm in front of the scaffolds center to measure the applied sound pressure. C) The tympanic membrane in the bony limbus was measured with LDV during a multi-sinusoidal signal excitation between 100 Hz and 5 kHz at a sound pressure level of about 90 dB SPL. A probe Microphone (ER-7C, Etymotic Research) was placed about 1-2 mm in front of the tympanic membrane center to measure the applied sound pressure.

TM characteristics

Macroscopically the human TM has an oval shape with a vertical diameter ranging from 8.5 mm to 11 mm and a horizontal diameter ranging from 8 mm to 9 mm (Figure S2A). It is conically shaped towards the malleus with a cone angle between 132° and 137° and a cone depth of 1.42-2 mm (Figure S2B and D).[1–4] Further, the TM is divided into two main areas, the *pars tensa* and the *pars flaccida*. The latter is thicker and relatively small (3.3 mm^2) in humans when compared to the *pars tensa* (62.1 mm^2).[5] The thickness distribution of the *pars tensa* differs, ranging from 30 µm to 150 µm. It exhibits a layered structure with a mucosal layer on the medial side, two collagen fiber layers in the center and an epidermal layer on the

lateral side (Figure S2C). The inner collagen fiber layer consists of circumferential fibers and the outer layer of radial collagen fibers.[4,6] This substructure of collagen fibers plays an important role for the sound transmission to the *malleus* and thus, the ossicular chain.[7,8]

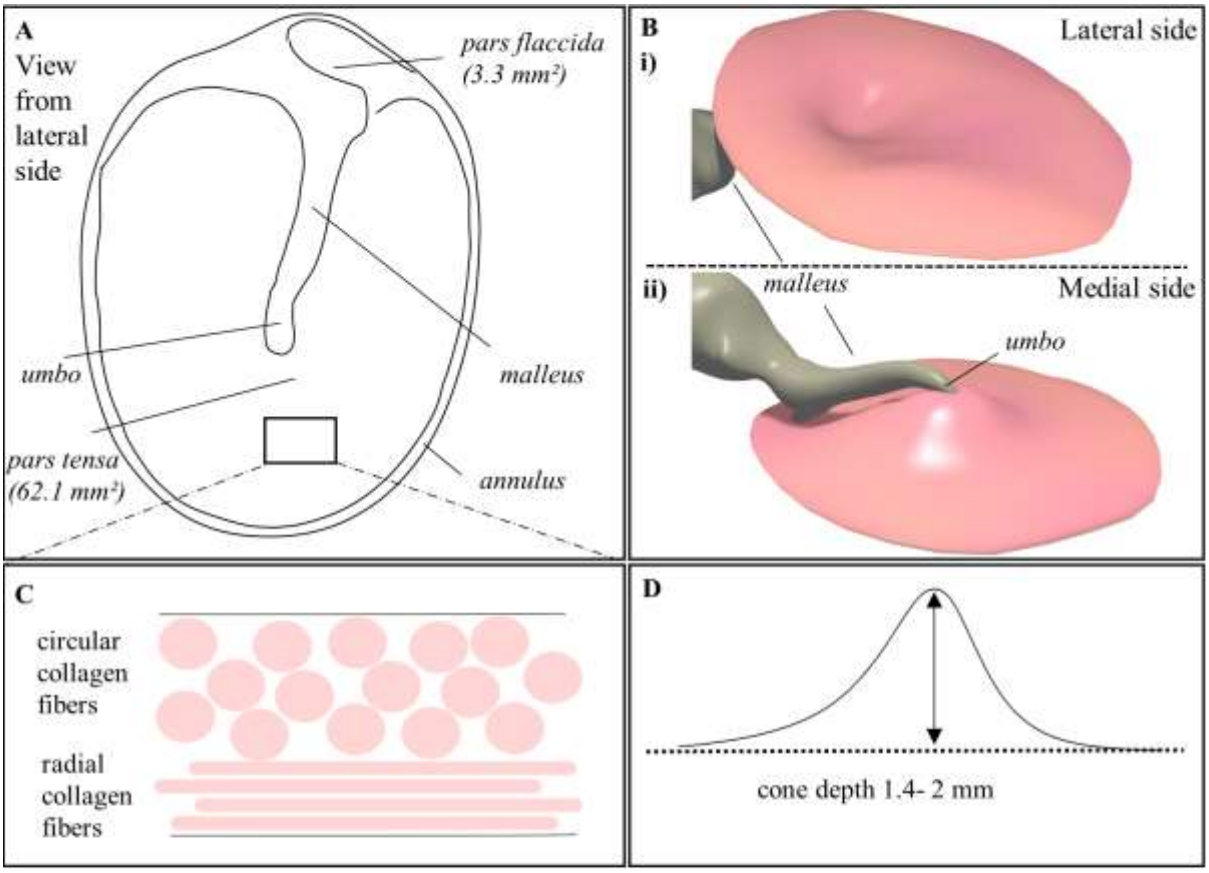

Figure S2. A) Sketch of the different parts and areas of the TM. B) 3D graphic of a human TM to show the conical shape. Bi) the TM faces the outer ear and Bii) the inner middle ear. C) Illustration of the layered structure of the circumferential and radial collagen fibers of the pars tensa. D) Sketch of the conical height of the TM.

Indentation measurements of the human tympanic membrane

After explantation, one human TM was placed within the experimental setup for mechanical investigation and fixed with minimum force possible to keep the TM in place. Four measurements were performed at different points of the membrane to characterize the response towards the applied mechanical load and are depicted in Figure S3. The measurements were performed close to the center of the membrane and showed a similar behavior to each other. Due to the conical shape of the TM an initial amount of pressure was

necessary to fully span the TM and as such, the steepest slope occurred later at a relative displacement between 1500% and 3000%, when compared to the flat MEW scaffolds.

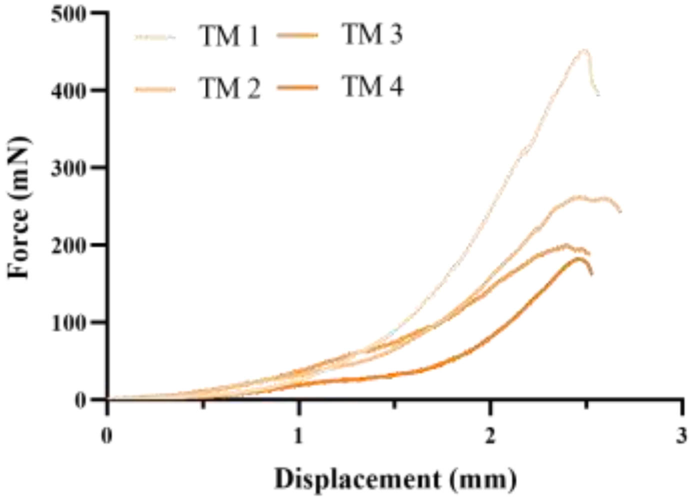

Figure S3. Four indentation measurements of one human TM at different positions.

Force/displacement graphs for MEW-Scaffolds

All indentation measurements carried out are presented in the following Figure S4. Each scaffold was deflected for at least 2 mm. The last row shows the differences resulting from the collagen coating of the MEW scaffolds as well as the cyclic loading measurements for 4L45d10w250 scaffolds w/ and w/o collagen.

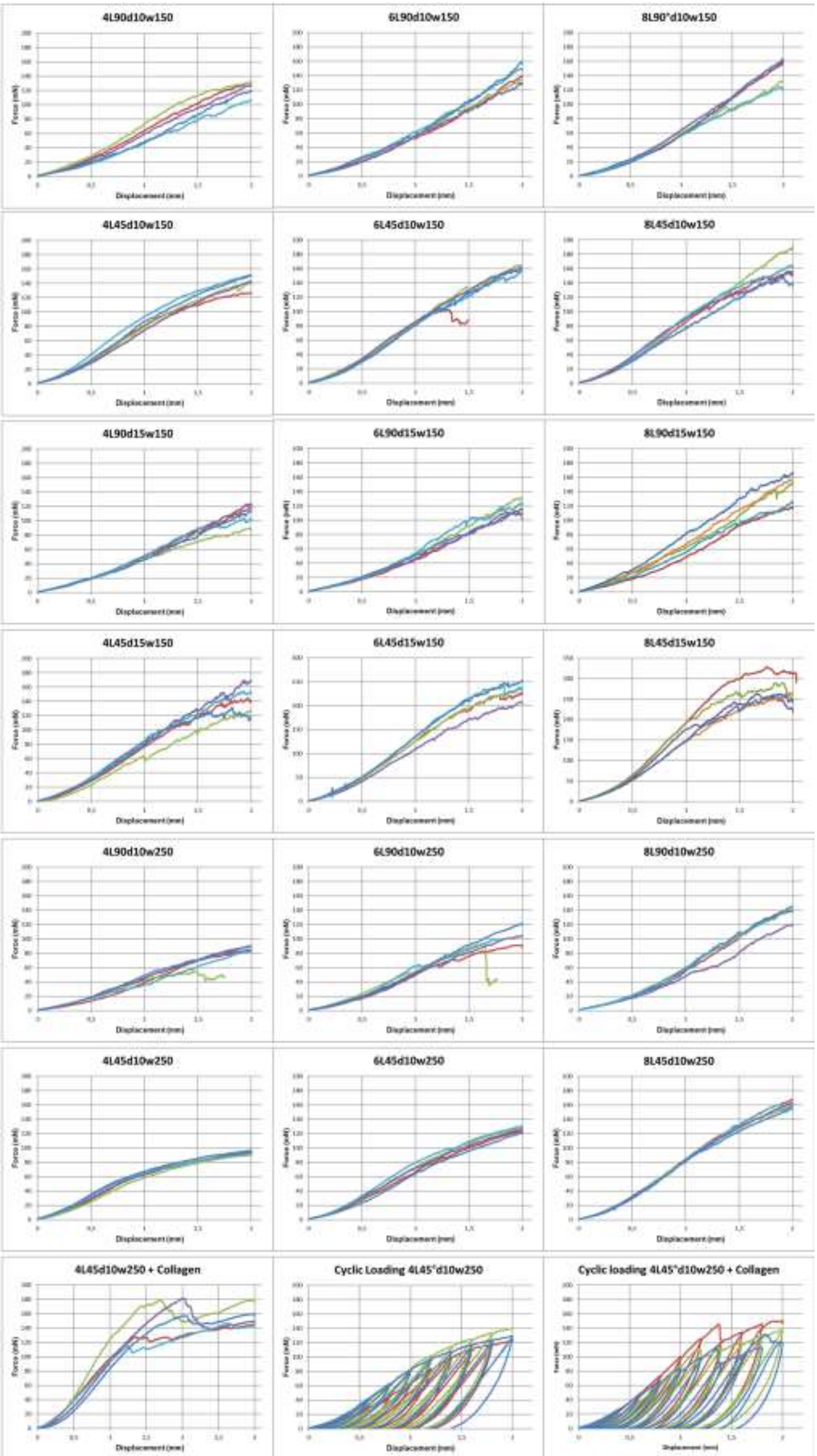

Figure S4. Individual indentation measurements of each investigated scaffold design.

Vibrational Measurements

Frequency response plots of the measured sound transfer function of the different scaffold designs are presented in Figure S5.

Compared to the test stand results described in chapter 2.1.1, the FR of the TMs in the temporal bones (without *malleus*) ranged from about 350 Hz to 850 Hz (geometric mean about 545 Hz). This showed that an explanted TM loses stiffness due to the loss of integrity in shape and again it shows the large range of anatomical variance.

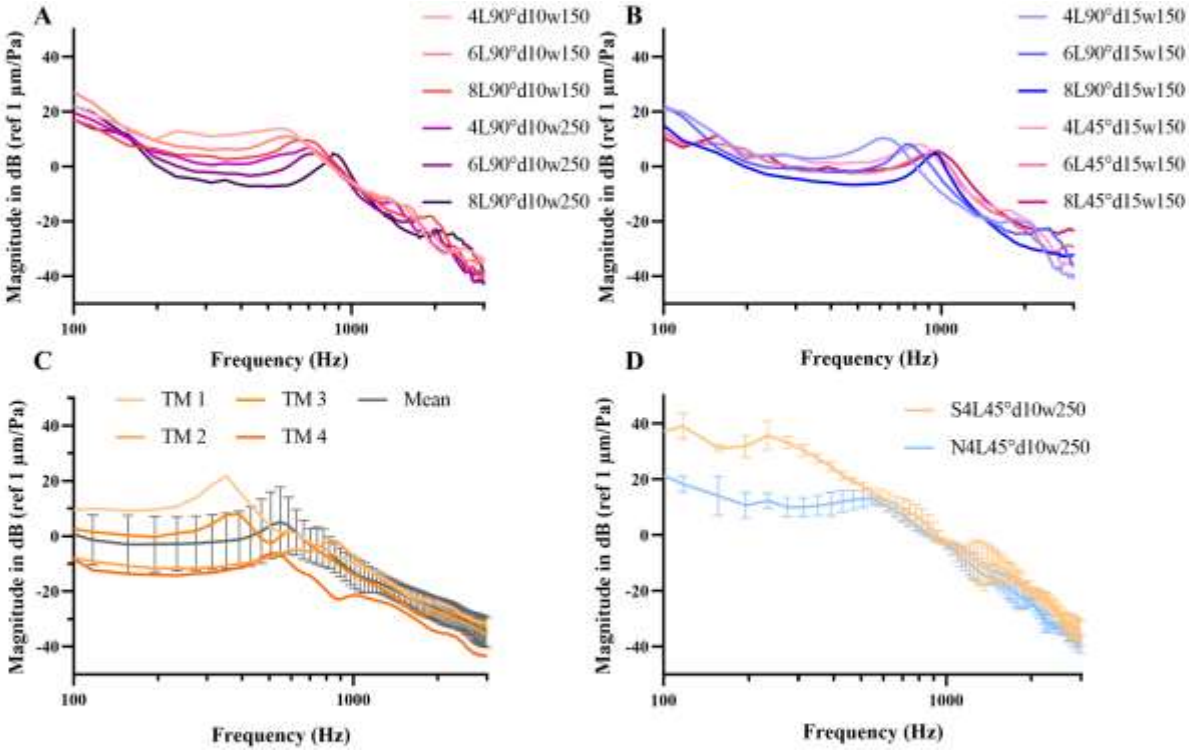

Figure S5. Additional data sets of all acoustically investigated MEW scaffold designs (mean values, n=5). A demonstrates the influence of the fiber spacing. B indicates the influence of the layer-to-layer orientation. C shows the results of the sound transfer functions of the four human TMs in temporal bones. D represents the influence when the lowest fixation force to the scaffold (S4L45°d10w250) or higher fixation force (N4L45°d10w250) are applied. The higher clamping force and increases the preloading of the scaffold. Increased preloading leads then to a stiffer scaffold, which transfers the sound with a lower amplitude and a higher resonance frequency occurs when compared to a scaffold fixed with lower force.

For keeping the curve characteristics of the TM sound transfer functions during averaging, the mean for the TM was calculated using a curve morphology preserving algorithm (see supplement paragraph *Algorithm for averaging the tympanic membrane measurements*).

Like the following paper states, at frequencies above 3 kHz the tympanic membrane vibrates chaotically. Hence, the most valuable information is acquired below 3 kHz. With focus on certain parts of the sound transfer properties, and partially restricted by the measurement technology, the frequency ranges shown were limited.[9] The scaffolds were investigated with single point LDV measurements. Thus, the more complex higher modes can't be detected in the center of the scaffolds.[10] Furthermore, the hearing range above the range of speech (up to 6 kHz) is not focus of investigations in case of implant hearing outcome (PTA = pure tone average measured at some specific frequencies, up to only 3 or 4 kHz).[11]

Evaluation of resonance frequency

The overall stiffness is mainly relevant for the sound transfer function characteristic (magnitude and first resonance frequency), in our case. In detail, the mass is more relevant with higher frequency; the stiffness is by theory mostly relevant for lower frequencies, like seen in the Figure S6. Our results outlined different results: The sound transfer, as we have shown in Figure 4, is strongly influenced by the architecture of the TM replacement. Furthermore, a relevant air-bone-gap of above 10 dB exists, which can persist in the whole frequency range (including low frequencies).[12]

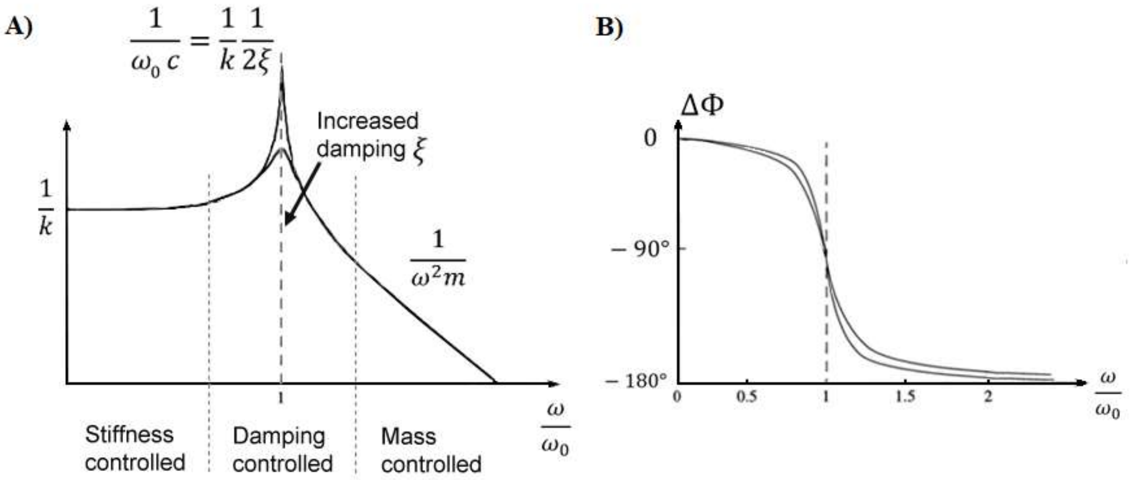

Figure S6. Display of the resonance frequency (dashed line) in A) at different damping states.[13] In B) a typical phase shift diagram at the resonance frequency (dashed line) is shown.[14]

For low frequencies, the equation (S1), which describes the frequency response function,

$$\frac{X}{F} = \frac{1}{k - \omega^2 m + i\omega c} \tag{S1}$$

leads to $\frac{1}{k}$, with displacement X, force F, stiffness coefficient k, the circular frequency ω, the mass m, the imaginary unit i and the viscous damping coefficient c. The range below the resonance frequency is therefore called “stiffness controlled”, whereas for higher frequencies the mass controlled range is dominated by the term $1/(\omega^2$ m). The complete hearing range is not relevant for our investigation, as discussed before.

The average vibration curve and the corresponding standard deviation of every investigated scaffold design is shown in Figure S7.

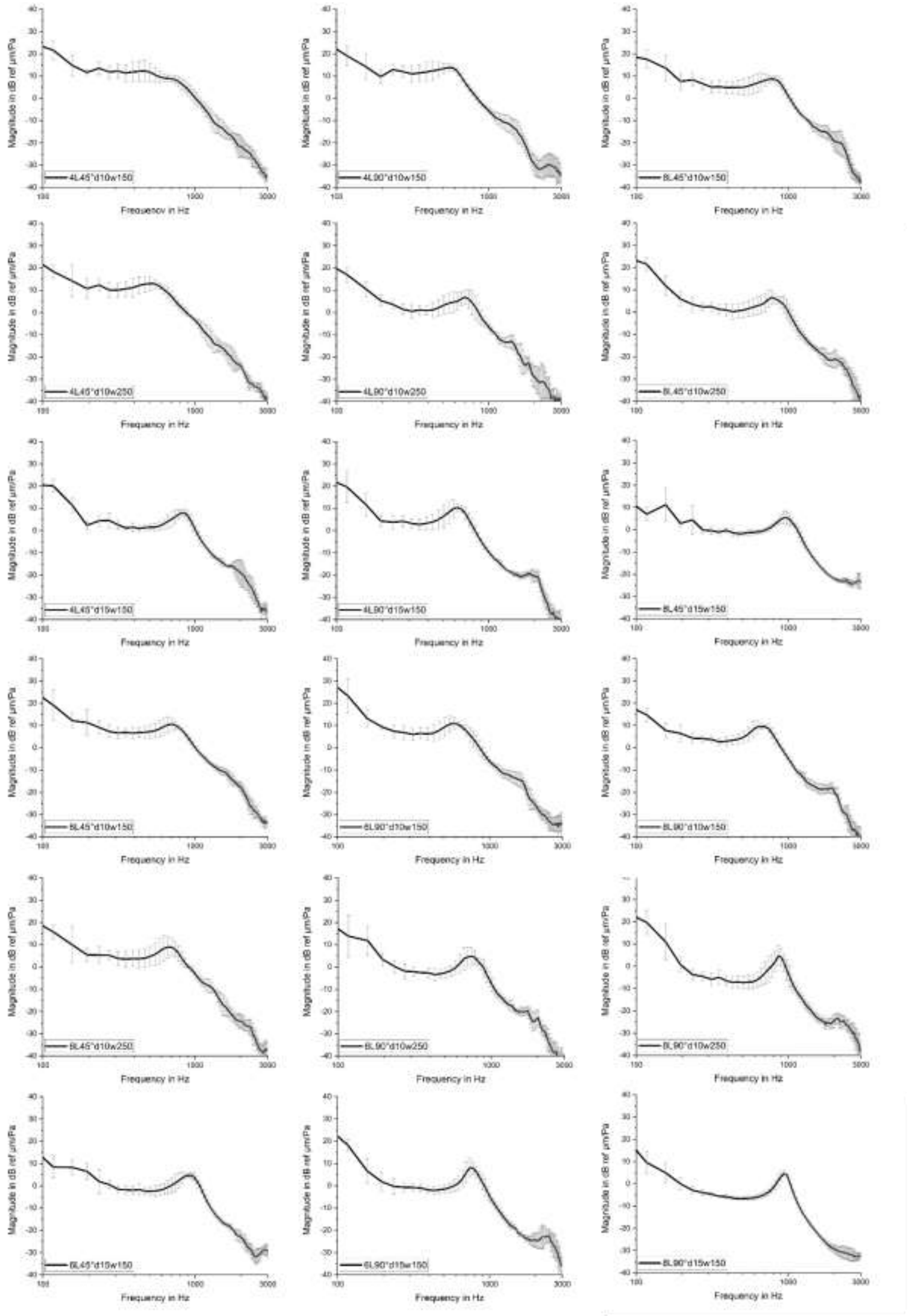

Figure S7. Single mean curves with standard deviation for each scaffold batch (n=5)

Collagen layer thickness

The thickness of the applied collagen coating was examined in vacuum and therefore in completely dried state by scanning electron microscopy and ranged from 300 nm to 400 nm (Figure S8).

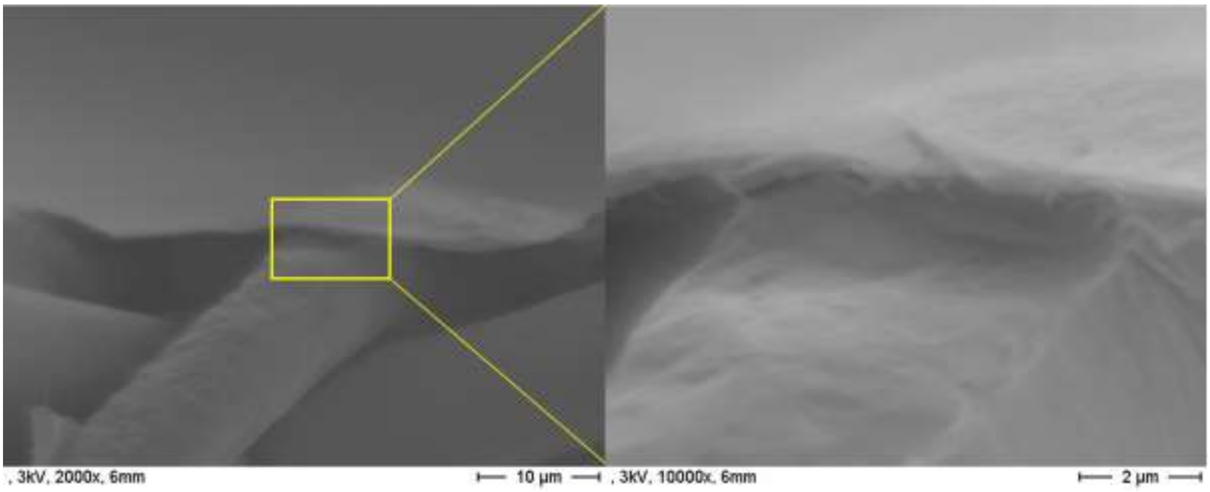

Figure S8**.** Scanning electron microscopy images showing the collagen layer on top of the 10 µm thick PCL fibers of a MEW scaffold. The collagen layer thickness ranged from 300 nm to 400 nm.

Vibration modes of TM and Scaffold

The first three vibration modes of a human TM and a collagen coated, melt electrowritten Scaffold with the 4L45°d10w250 design are shown in Videos Figure ***9*** (the videos need to be separately downloaded).

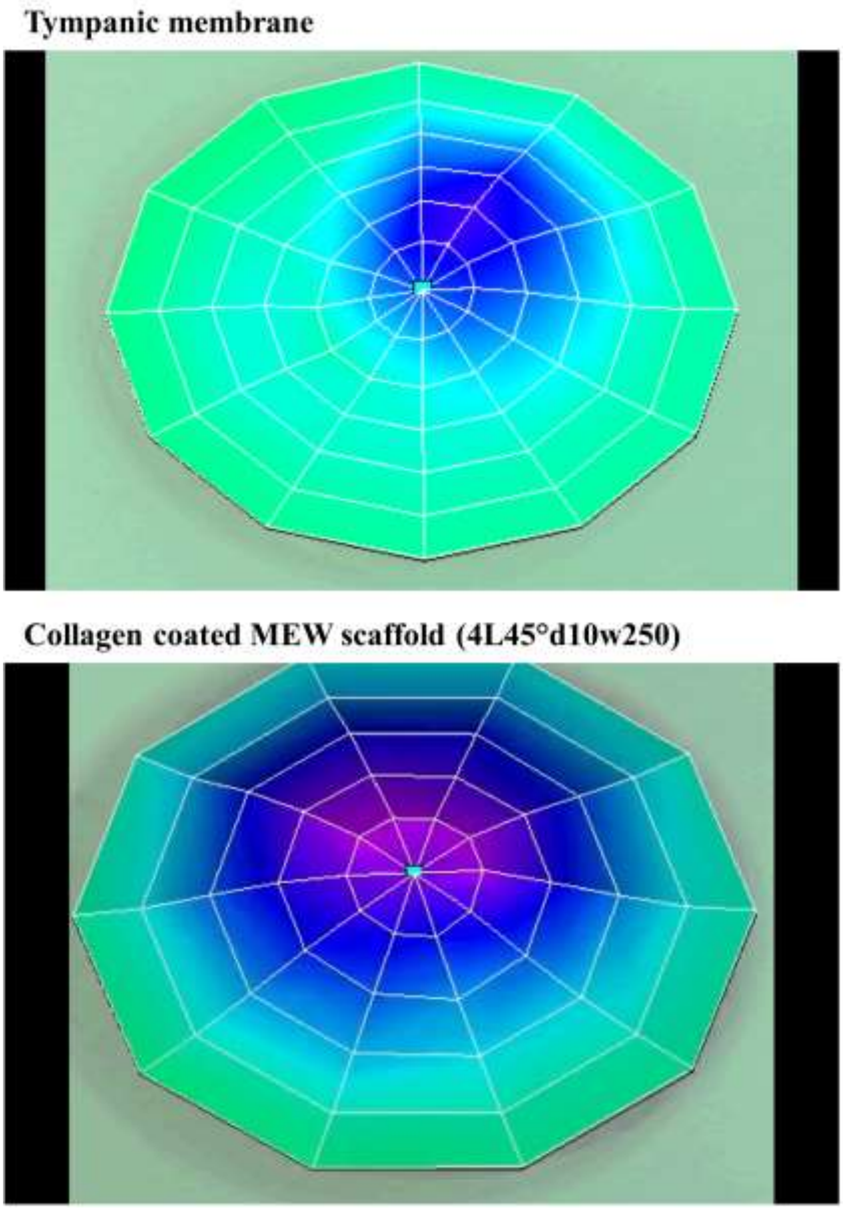

Videos Figure 9. The first video (vibrationModes_TM.avi) shows the first three vibration modes of a human tympanic membrane. The other video (vibrationModes_MEW.avi) exhibits the first three vibration modes of a collagen coated MEW scaffolds with the 4L45°d10w250 design.

UV-light treatment of the collagen coating

Scaffolds w/ and w/o collagen were treated for 2 h with UV light (λ = 254 nm) to crosslink the collagen. Afterwards they were seeded with keratinocytes (human cell line HaCaT) to assess their cellular response. Live/Dead and DAPI/Phalloidin stainings were performed and are shown in

Figure *S10*. An impact of UV-light on the cell behavior was not observed within the microscopic images.

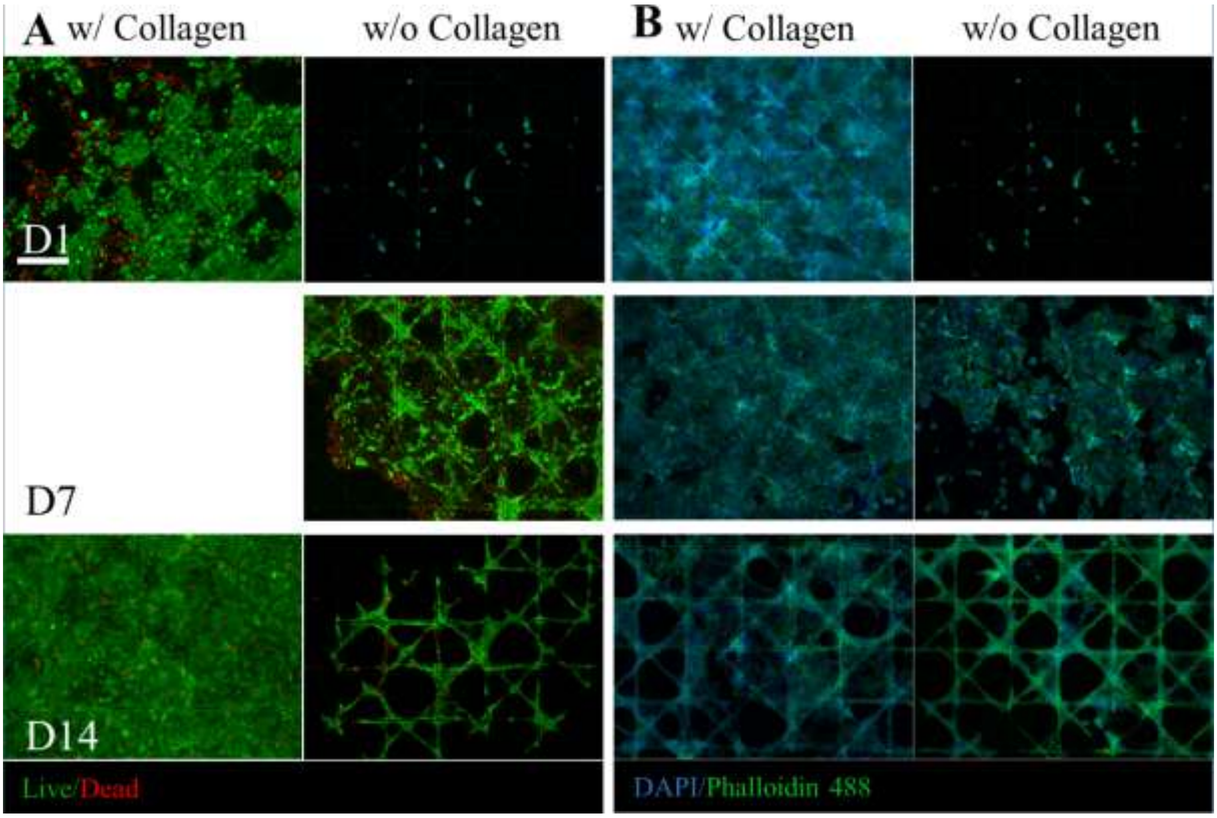

Figure S10. Live/Dead and DAPI/Phalloidin staining of UV light treated scaffolds w/ and w/o collagen for assessment of. biological activity over a time of 14 days. x10 magnification Scale bars: 250 µm;

Algorithm for averaging the tympanic membrane measurements

For preserving the curve morphology of the TM sound transfer functions, an averaging algorithm based on Gladline et al.[15] and James et al.[16] was utilized and written in Matlab (R2018b, 64-bit, MathWorks Inc., USA). In detail, one landmark, the first resonance frequency, was chosen for warping the measured TM curves. In this way, the mean curve was oriented at the geometric average of the first resonance frequencies of the single curves. For this, the single first resonance frequencies were determined manually.

Brief description of the algorithm approach:

Origin (OriginPro 2019, 64-bit., OriginLab Corp., USA)

1. Logarithmic calculation of the sound transfer function into dB ref 1 µm/Pa with 20*lg(0.001 * nm/Pa).

2. Manual selection of landmark points by determination of the resonance peak and the corresponding phase change.
3. Interpolation of data to get same database for x (Frequency range from about 40 Hz to 2070 Hz)
4. Export data for processing in Matlab

Matlab

5. Calculation of the geometric mean of the first resonance frequency
6. Define weights and algorithm step size for iterative processing (calculate frequency warping function and cost function with brute force approach)
7. Process curve registration by calculating frequency warping function for each weight set and calculate cost function for each single sound transfer function curve
8. Load result file with minimum values and choose weights with minimum cost function value for each curve
9. Choose the weight set with the minimum value of the cost function for each single sound transfer function curve
10. Calculate optimum warping function, plot and export.

Algorithm results for TM curves in respect of Gladline et al.[15]:

Single frequencies in Hz oHG for the four TM:
[351; 859; 390; 564]

B-Spline knots:
knots = [39, 39, 39, 200, 500, 976, 2070.3, 2070.3, 2070.3];

weights for the single warping functions:
weights=[-6 -2 -2 4 0,
-2 0 4 -6 0,
-6 -6 4 -2 0,
-6 2 0 -2 0];

Weight step size for optimization iterations:
resolution = 2;
bottomBorder = -6;

topBorder = 6;

Single frequencies in Hz MS:

[312; 469; 156; 195];

B-Spline knots:

knots = [39,39,39,100,400,2070.3,2070.3,2070.3];

weights for the single warping functions:

weights=[-4 6 1 6,

-4 4 -6 2,

-6 2 -2 2,

-5 2 -3 2];

Weight step size for optimization iterations:

resolution = 1;

bottomBorder = -6;

topBorder = 6;

[1] M. Ferrazzini, Virtual Middle Ear: A Dynamic Mathematical Model Based on the Finite Element Method, ETH Zurich, **2003**.
[2] F. Zhao, T. Koike, J. Wang, H. Sienz, R. Meredith, *Med. Eng. Phys.* **2009**, *31*, 907.
[3] R. Z. Gan, B. Feng, Q. Sun, *Ann. Biomed. Eng.* **2004**, *32*, 847.
[4] N. P. Daphalapurkar, C. Dai, R. Z. Gan, H. Lu, *J. Mech. Behave. Biomed.* **2009**, *2*, 82.
[5] W. F. Decraemer, W. R. J. Funnell, in *Chronic Otitis Media*, Kugler Publications, **2008**, pp. 51–80.
[6] H. Luo, C. Dai, R. Z. Gan, H. Lu, *J. Biomech. Eng.* **2009**, *131*, 1.
[7] G. Volandri, F. Di Puccio, P. Forte, C. Carmignani, *J. Biomech* **2011**, *44*, 1219.
[8] K. N. O'Connor, M. Tam, N. H. Blevins, S. Puria, *The Laryngoscope* **2008**, *118*, 483.
[9] J. P. Fay, S. Puria, C. R. Steele, *Proc. Natl. Acad. Sci.* **2006**, *103*, 19743.
[10] J. T. Cheng, A. A. Aarnisalo, E. Harrington, M. del S. Hernandez-Montes, C. Furlong, S. N. Merchant, J. J. Rosowski, *Hear. Res.* **2010**, *263*, 66.
[11] Committee on Hearing and Equilibrium, *Otolaryngol. Head Neck* **1995**, *113*, 186.
[12] M. Berglund, S. Olaison, Å. Bonnard, M. Fransson, M. Hultcrantz, R. Florentzson, C. Dahlin, P. O. Eriksson, E. Westman, *Clin. Otolaryngol.* **2020**, *45*, 357.
[13] Rossing, Thomas D., Fletcher, Neville H., *Principles of Vibration and Sound*, Springer Verlag, New York, **2004**.
[14] L. F. Braña, in *Quiet Transformers: Design Issues*, Baiona, Spain, **2013**.
[15] K. Gladiné, J. J. J. Dirckx, *Hear. Res.* **2018**, *363*, 39.
[16] G. M. James, *Ann. Appl. Stat.* **2007**, *1*, 480.
